# Comparison of chitin‐induced natural transformation in pandemic *Vibrio cholerae* O1 El Tor strains

**DOI:** 10.1111/1462-2920.15214

**Published:** 2020-09-09

**Authors:** Sandrine Stutzmann, Melanie Blokesch

**Affiliations:** ^1^ Laboratory of Molecular Microbiology, Global Health Institute School of Life Sciences, Ecole Polytechnique Fédérale de Lausanne (EPFL) Lausanne CH‐1015 Switzerland

## Abstract

The human pathogen *Vibrio cholerae* serves as a model organism for many important processes ranging from pathogenesis to natural transformation, which has been extensively studied in this bacterium. Previous work has deciphered important regulatory circuits involved in natural competence induction as well as mechanistic details related to its DNA acquisition and uptake potential. However, since competence was first reported for *V. cholerae* in 2005, many researchers have struggled with reproducibility in certain strains. In this study, we therefore compare prominent seventh pandemic *V. cholerae* isolates, namely strains A1552, N16961, C6706, C6709, E7946, P27459, and the close relative MO10, for their natural transformability and decipher underlying defects that mask the high degree of competence conservation. Through a combination of experimental approaches and comparative genomics based on new whole‐genome sequences and *de novo* assemblies, we identify several strain‐specific defects, mostly in genes that encode key players in quorum sensing. Moreover, we provide evidence that most of these deficiencies might have recently occurred through laboratory domestication events or through the acquisition of mobile genetic elements. Lastly, we highlight that differing experimental approaches between research groups might explain more of the variations than strain‐specific alterations.

## Introduction

The causative agent of cholera, *Vibrio cholerae*, is an interesting model organism for studying dual bacterial lifestyles due to its ability to thrive both in the human gut and in natural aquatic habitats (Colwell, 1996; Nelson *et al*., 2009). However, only a limited set of *V. cholerae* strains is responsible for cholera pandemics, while a broad variety of isolates do not cause the disease and are primarily found in the environment. Therefore, to better understand pathogen emergence, it is important to understand basic differences between pathogenic and environmental strains, as well as the capability of different species for the horizontal acquisition of genetic material.

In its environmental reservoir, *V. cholerae*'s attachment to biotic surfaces often results in biofilm formation and the degradation of the underlying nutritious surfaces, such as the chitin polymer. Chitin is the most abundant polysaccharide in aquatic environments and the primary carbon source for many marine bacteria (Gooday, 1990), including most Vibrios (Hunt *et al*., 2008; Le Roux and Blokesch, 2018). Apart from its role as a major carbon source, in *V. cholerae*, chitin also triggers natural competence for transformation (Meibom *et al*., 2005), which is the state in which the bacterium is able to take up free DNA and incorporate it into its genome by double homologous recombination. As a mechanism of horizontal gene transfer (HGT), natural competence enables *V. cholerae* (Meibom *et al*., 2005) and other *Vibrio* species (Gulig *et al*., 2009; Pollack‐Berti *et al*., 2010; Metzger *et al*., 2019) to exchange DNA stretches including those that harbour genes or operons encoding new pathogenic or metabolic traits (Blokesch and Schoolnik, 2007; Miller *et al*., 2007).

**Fig. 1:**
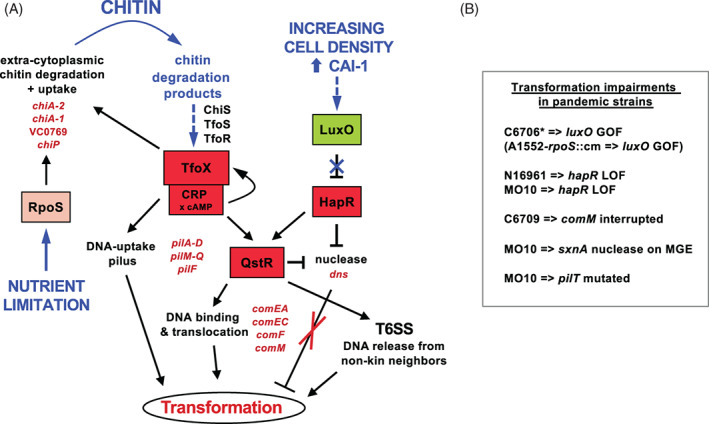
The competence regulatory circuit in *V. cholerae*. A. Competence induction in *V. cholerae* is dependent on chitin induction and quorum sensing (mostly driven by the species‐specific autoinducer CAI‐1). The scheme is an updated version of the model proposed by Meibom *et al*. (2005) (see Supporting Information Fig. S1). Red boxes denote positive regulators and the most important regulated genes are shown in red writing. For details see text. B. Causation of impaired transformation in a subset of pandemic strains. GOF, gain‐of‐function; LOF, Loss‐of‐function.

Since the initial discovery of chitin‐induced competence in *V. cholerae* (Meibom *et al*., 2005), several studies have addressed the underlying regulatory circuits and mechanistic aspects of the DNA uptake process (Fig. 1). In addition to inducing the competence state, which includes the production of the DNA‐uptake complex (Seitz and Blokesch, 2013), chitin also induces the type VI secretion system (T6SS), a molecular killing device (Cianfanelli *et al*., 2016), in pandemic *V. cholerae* strains (Borgeaud *et al*., 2015). The T6SS fosters killing of adjacent bacteria in a contact‐ and kin‐discriminatory manner, thereby serving as a DNA acquisition system that enhances HGT (Borgeaud *et al*., 2015; Metzger and Blokesch, 2016; Veening and Blokesch, 2017). We recently showed that this link between neighbour predation and prey‐released DNA uptake allows long stretches of DNA, frequently exceeding 100 kilobases (kb) in length, to be horizontally transferred. (Matthey *et al*., 2019). Given the massive potential of T6SS‐mediated neighbour predation and its coupling to the concomitantly produced DNA‐uptake machinery, it is not surprising that the bacteria have evolved sophisticated regulatory circuits to control the onset of the competence state.

An essential inducer of competence in *V. cholerae* (Meibom *et al*., 2005), chitin is degraded by extracellular chitinases that are either produced by moulting zooplankton, by other bacteria through ‘sloppy eating’, or by starved *Vibrio* species themselves (Keyhani and Roseman, 1999). Chitinases predominantly release *N*‐acetyl‐glucosamine (GlcNAc) in the form of dimers or trimers from the chitin polymer, whereas monomeric GlcNAc is not a primary cleavage product (Gooday, 1990; Li and Roseman, 2004). Diacetylchitobiose, the GlcNAc dimer (also frequently called chitobiose), acts as a strong chemoattractant for diverse *Vibrio* species (Bassler *et al*., 1989) and is subsequently taken up via an ABC‐type transporter encoded by the genes VC0619–VC0616 in *V. cholerae* in conjunction with the periplasmic chitin‐binding protein Cbp (VC0620), thereby avoiding carbon catabolite repression (Li and Roseman, 2004; Blokesch, 2012b). The chitobiose is further degraded into monomeric sugars and finally shuttled into central metabolism (Gooday, 1990; Hunt *et al*., 2008).

Understanding of the signalling pathways that link chitin sensing to competence induction has significantly improved in recent years (Fig. 1 compared to Supporting Information Fig. S1). The chitin catabolic cascade (e.g., the chitobiose catabolic operon, VC0620–VC0611; (Li and Roseman, 2004) is mostly controlled by ChiS, a noncanonical, membrane‐embedded, one‐component sensor kinase (Klancher *et al*., 2020a). This pathway is important for chitin catabolism, while the sensing of chitobiose by yet another noncanonical transmembrane regulator named TfoS is, together with its regulated small RNA TfoR (Yamamoto *et al*., 2011; Dalia *et al*., 2014; Yamamoto *et al*., 2014), essential for the production of the major regulator of competence, TfoX (Meibom *et al*., 2005). Artificial TfoX production suppresses the chitin requirement and triggers competence induction even under rich medium conditions if catabolite repression does not occur (Meibom *et al*., 2005; Blokesch, 2012b). Because TfoX proteins are often highly conserved, their homologous or even heterologous production induces competence in diverse *Vibrio* species such as *V. cholerae*, *V. parahaemolyticus*, *V. alginolyticus*, *V. fischeri*, *V. campbellii* and *V. vulnificus* (Pollack‐Berti *et al*., 2010; Metzger *et al*., 2016; Metzger *et al*., 2019; Simpson *et al*., 2019).

In addition to chitin‐induced signalling through TfoX, competence induction in *V. cholerae* also relies on input from the bacterium's quorum sensing (QS) system (Meibom *et al*., 2005), which is controlled by the high cell density master QS regulator HapR (Mukherjee and Bassler, 2019). Chitin signalling and QS pathways are linked by an intermediate regulatory protein, which we named QS‐ and TfoX‐dependent regulator QstR (Lo Scrudato and Blokesch, 2013). QstR is a dual transcription factor of the LuxR‐type family (Jaskólska *et al*., 2018) that induces a subset of competence genes (such as *comEA*, *comEC*, *comF*, and *comM*; recently reviewed by (Dubnau and Blokesch, 2019). In addition to *qstR* and therefore competence gene induction, HapR plays another important role in natural transformation, namely as a repressor of the periplasmic and extracellular nuclease Dns. Previously, we showed that Dns degrades transforming material in QS‐defective strains and therefore impairs transformation (Blokesch and Schoolnik, 2008). In wild‐type cells, the encoding gene *dns* is, however, repressed by direct binding of HapR, a repression that is further enhanced by QstR (Blokesch and Schoolnik, 2008; Lo Scrudato and Blokesch, 2013; Jaskólska *et al*., 2018) (Fig. 1).

While the past 15 years since its discovery (Meibom *et al*., 2005) have significantly advanced our knowledge of competence regulation and DNA uptake of chitin‐induced naturally competent *V. cholerae* (Metzger and Blokesch, 2016; Dubnau and Blokesch, 2019), many questions remained unanswered. Moreover, selective findings or their interpretation often differ between studies from various groups, which is frequently justified by the use of different *V. cholerae* patient isolates. The reasons for such strain‐specific responses could be due to differences in the underlying regulatory circuits or laboratory domestication events, making the results less biologically meaningful. For instance, it is well known that the QS pathway is prone to mutations in the *hapR* and *luxO* genes (Joelsson *et al*., 2006; Jung *et al*., 2015; Stutzmann and Blokesch, 2016), with LuxO usually acting as a repressor of the *hapR* transcript via the Qrr sRNAs at low cell density (Mukherjee and Bassler, 2019). In this context, Kimbrough and Stabb showed that prolonged stationary phase conditions (as could occur when keeping Vibrios too long on plates) caused QS‐impairing gain‐of‐function (GOF) mutations in *luxO* of *V. fischeri* (proteins named LuxO*; Kimbrough and Stabb, 2015). A similar GOF mutation in *luxO*, which resulted in the G333S amino acid exchange in LuxO, led to an impaired high cell density response in the pandemic strain C6706 (Stutzmann and Blokesch, 2016). This C6706 (Str^R^) strain was subsequently shared among many laboratories, causing irreproducibility issues.

In this study, we compared the chitin‐induced competence and transformation behaviour in several of the most prominent pandemic O1 El Tor *V. cholerae* isolates and the close relative O139 strain MO10. Through comparative analysis of *de novo* assembled genomes of strains A1552, N16961, C6706, C6709, E7946, and P27459 based on long‐read, whole‐genome, PacBio sequencing data combined with experimental validation, we show that the competence regulon is completely conserved. We also provide evidence that all strains with a fully functional quorum sensing circuit behave in a highly comparable manner with respect to competence induction and natural transformability, which suggests that previous studies differed due to experimental differences and not due to extensive strain variations. Moreover, we found that *comM* of strain C6709 is interrupted by a mobile genetic element and show that this interruption impairs the strain's natural transformability.

## Results and discussion

### The primary role of CAI‐1 in competence induction is conserved among pandemic strains

**Fig. 2:**
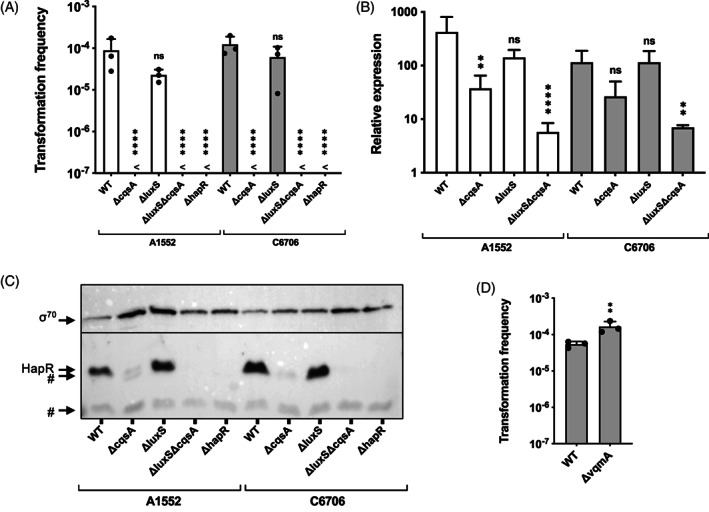
Absence of the species‐specific CAI‐1 severely impacts transformation in monocultures. *V. cholerae* strains lacking the autoinducer synthases CqsA (for CAI‐1) and/or LuxS (for AI‐2) were tested for their natural transformability on chitin (A), their *hapR* transcripts (B), and their HapR protein levels (C). Wild‐type (WT) and *hapR*‐minus (∆hapR) strains served as controls. The experiments were performed with mutants in two different strain backgrounds (A1552 and C6706). Panels (A) and (D): Transformation was tested on chitin surfaces and transformation frequencies are given on the *Y*‐axis. Panel (D) shows that the VqmA‐DPO QS system is dispensable for natural transformation. Panel (C): Sigma70 detections served as a loading control. The known cross‐reacting bands of the Anti‐HapR antibodies are indicated by # in the Western blot analysis. All experiments were performed three independent times and the mean values are shown for all graphs (±SD). <, below detection limit. Statistical analysis compared each mutant to their parental WT strains. Statistical analyses on the log‐transformed data: (A, B) one‐way ANOVA with a Sidak's multiple comparisons test; (D) two‐tailed *t*‐test. The detection limit was used for statistical analysis for conditions in which no transformants were recovered. ** *P* < 0.01; ****P < 0.0001; ns, not significant.

Competence regulation in *V. cholerae* has been extensively studied, and strain‐specific responses have frequently been reported, even for patient isolates representing the current ongoing seventh pandemic cholera. In contrast to environmental isolates, these pandemic strains are thought to be highly similar if not clonal, at least with respect to the core genome (Mutreja *et al*., 2011). We therefore aimed to first compare the impact of QS on competence induction and transformation, using two prominent *V. cholerae* isolates, namely strains A1552 and C6706, both of which belong to the Latin American clade of seventh pandemic strains (Blokesch, 2012c; Domman *et al*., 2017) (Supporting Information Table S1). We previously showed that the competence and transformation of strain A1552 was primarily driven by the species‐specific cholera autoinducer 1 (CAI‐1), while the effect of the more universal autoinducer AI‐2 (Ng and Bassler, 2009) seemed negligible in this context (Suckow *et al*., 2011). In the absence of the CAI‐1 synthase CqsA, natural transformation was mostly abrogated or very close to the detection limit, while the absence of the AI‐2 synthase LuxS did not significantly impact the bacteria's transformability. The absence of both autoinducer synthases reflected the *hapR*‐deficient phenotype that lacked transformability (Suckow *et al*., 2011; Lo Scrudato and Blokesch, 2012). Using strain C6706, Antonova and Hammer came to slightly different conclusions (Antonova and Hammer, 2011). They found that while a *hapR* mutant was likewise non‐transformable, both *cqsA*‐minus and surprisingly *cqsA/luxS*‐double mutants were still significantly transformable (Antonova and Hammer, 2011). To address this conflicting result, we repeated these transformation experiments with the two strains A1552 and C6706 and their derivatives (C6706 mutants kindly provided by B. Hammer) in parallel using an optimized transformation protocol based on chitin flakes as the competence inducer (Marvig and Blokesch, 2010 and Method section). As shown in Fig. 2, the direct side‐by‐side comparison resulted in almost identical transformation phenotypes for both pandemic strains and their autoinducer synthase single or double mutants.

To better understand the impact of the autoinducer synthases on QS, and therefore natural competence, we next tested the strains' *hapR* transcript levels using quantitative reverse transcription PCR (qRT‐PCR). We saw reduced *hapR* transcript levels for those strains that lacked the CAI‐1 synthase and a highly significant *hapR* transcription reduction for the strains that lacked both autoinducer synthases (∆luxS∆cqsA; Fig. 2B). As *hapR* is known to be post‐transcriptionally regulated by several Qrr sRNAs, we also checked its protein production by western blotting. These data showed a highly significant reduction in HapR protein levels in CAI‐synthase mutants of strain A1552 and C6706 (∆cqsA), while the AI‐2 synthase mutants (∆luxS) retained high HapR levels (Fig. 2C). Importantly, no HapR was detectable in the absence of *cqsA* and *luxS*, no matter which pandemic strain background was tested (Fig. 2C). We therefore conclude that CAI‐1 plays a primary role in competence regulation in *V. cholerae*. This result supports our previous work showing the strong dominance of the species‐specific autoinducer CAI‐1 over the universal autoinducer AI‐2 with respect to natural competence and transformation of *V. cholerae* in monoculture (Suckow *et al*., 2011; Lo Scrudato and Blokesch, 2012). Furthermore, this result is in line with a more recent study on a similar species‐specific repression of the chitobiose utilization locus (Klancher *et al*., 2020b).

Since competence induction is linked to a high cell density state, we wondered if the newly identified VqmA‐DPO QS system of *V. cholerae* was also involved in natural transformation. Like HapR, the transcription factor VqmA (Liu *et al*., 2006), upon binding to the cognate autoinducer 3,5‐dimethylpyrazin‐2‐ol (DPO), indirectly represses biofilm formation (Papenfort *et al*., 2017), suggesting that both QS pathways might share common gene targets. However, testing a *vqmA* mutant for its natural transformability showed that the system is not essential for transformation (Fig. 2D).

### The alternative sigma factor RpoS does not foster HapR protein production

**Fig. 3:**
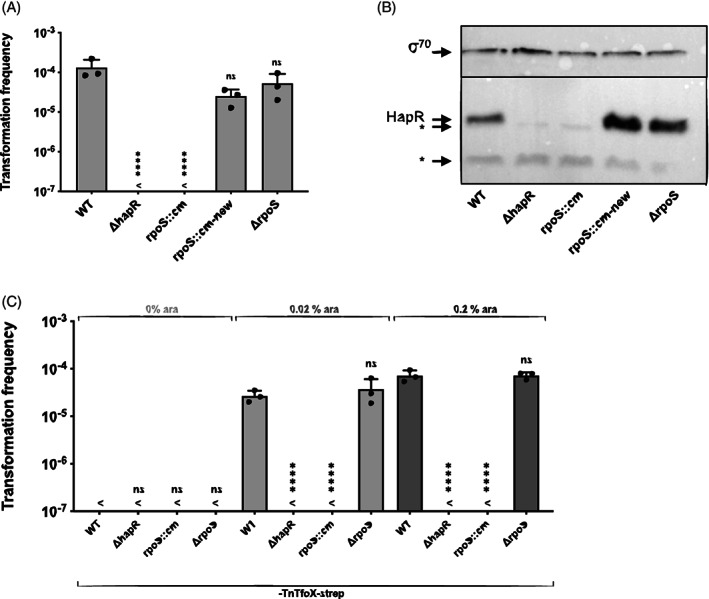
RpoS‐deficient *V. cholerae* are producing HapR and are naturally transformable. (A and C) WT and *rpoS*‐or *hapR*‐minus strains were tested for natural transformation under chitin‐dependent (A) or chitin‐independent (C) conditions. Details for (A) as in Fig. 2A. B. Strains for HapR detection by Western blotting were grown in plain LB medium and processed as those samples in Fig. 2C. For panel (C), the strains carried inducible TfoX on a transposon and were grown in LB medium in the absence (0%), or presence (0.02% or 0.2%) of arabinose as an inducer as indicated above the graph. All experiments were done three independent times and their average is shown in the bar graphs (±SD). <, below detection limit. Statistical analyses: (A) one‐way ANOVA with Tukey's multiple comparisons test. The detection limit was used for statistical analysis for conditions in which no transformants were recovered. ****P < 0.0001; ns, not significant.

The data shown above confirmed the primary role of HapR in competence induction and nuclease repression, so we therefore sought to investigate the production of HapR itself. In the first publication on chitin‐induced natural competence by the Schoolnik laboratory, the authors showed that a *rpoS* mutant of *V. cholerae* was non‐transformable and suggested that this phenotype might be based on RpoS's effect on *hapR* expression (Meibom *et al*., 2005). Previous work had suggested that RpoS was required for *hapR* transcription, since an *rpoS* mutant showed reduced *hapR* transcript levels (Yildiz *et al*., 2004) and decreased HA/protease (HapA) activity (Yildiz and Schoolnik, 1998) (the encoding hapA gene is positively regulated by HapR). However, recent work challenged this interpretation by showing that RpoS did not affect QS‐dependent competence regulation and instead exerted its effect via the production of extracellular chitinases, therefore using the chitin‐induction branch of the competence network (Dalia, 2016) (Fig. 1). We therefore repeated the initial experiment that was reported from the Schoolnik laboratory (Meibom *et al*., 2005; co‐authored by the senior author of this study), and compared the WT, *hapR*‐, and *rpoS*‐minus strains for their natural transformability on chitin, which confirmed the absence of transformants for the two mutants (Fig. 3A). Moreover, both mutants remained non‐transformable even upon chitin‐independent TfoX production (Fig. 3C; *rpoS::cm*). Given these conflicting results compared to the convincing data provided by Dalia (Dalia, 2016), we genetically reengineered the *rpoS* mutant in two ways: first, by transferring the inactivated *rpoS* allele containing a chloramphenicol‐resistance cassette from the original *rpoS* mutant strain (*rpoS::cm* corresponding to FY1; Yildiz and Schoolnik, 1998; Meibom *et al*., 2005) into the WT strain via natural transformation (mutant *rpoS::cm‐*new); and second, by designing a completely new in‐frame *rpoS* deletion strain (mutant ∆rpoS) using natural transformation coupled to flip recombination (TransFLP; Marvig and Blokesch, 2010; Silva and Blokesch, 2010; Blokesch, 2012a). Surprisingly, both of these new mutants were naturally transformable on chitin at levels that were statistically insignificant from the parental WT strain (Fig. 3A). Consistently, we also showed that the in‐frame deleted *rpoS* mutant was fully transformable under TfoX‐producing conditions in rich medium (Fig. 3C).

These results generated two new questions, namely (i) why the original *rpoS* mutant was non‐transformable while the newly engineered mutants, which were fully verified for the absence of *rpoS* by PCR and Sanger sequencing, were fully transformable; and (ii) why the results under chitin‐inducing conditions differed compared to Dalia's work (Dalia, 2016). To address the first question, and given that the original *rpoS* mutant phenocopied several HapR‐deficient phenotypes (Yildiz and Schoolnik, 1998), we first sequenced the strain's *hapR* and *luxO* genes. While the *hapR* gene was identical to the gene in the parental A1552 strain, *luxO* had acquired a point mutation at base pair 359 (base pair 317 according to the initial *luxO* ORF annotation; (Heidelberg *et al*., 2000). This mutation resulted in a valine‐to‐alanine exchange of amino acid 120 (V120A; V106A according to the old *luxO* annotation), which is identical to the LuxO*[V106A] GOF mutation in *V. fischeri* (Kimbrough and Stabb, 2015). Given this finding, we next tested all *rpoS* mutants for their HapR production and showed the absence of this transcription regulator in the original *rpoS::cm* mutant, consistent with a LuxO* GOF phenotype (Fig. 3B). Importantly, both newly engineered *rpoS* mutants produced copious amounts of HapR at high cell densities (Fig. 3C), challenging the general notion that RpoS positively regulates HapR production (Yildiz *et al*., 2004; Conner *et al*., 2016). At this point, we cannot distinguish whether the GOF *luxO* mutation was introduced into the *rpoS::cm* mutant strain when it was initially generated (Yildiz and Schoolnik, 1998) or whether it appeared through extended lab domestication while the strain was transferred between group members in the Schoolnik laboratory (we confirmed the *luxO* mutation in three independent aliquots that were transferred from the Schoolnik laboratory to the Blokesch laboratory in 2009). However, this *luxO* mutation could explain the initial observation by Yildiz and Schoolnik, who showed that the *rpoS::cm* mutant could be fully complemented for RpoS protein production, but only poorly compensated for the lack of HA/protease production and/or its secretion (Yildiz and Schoolnik, 1998).

### Contribution of RpoS to the expression of the chitin utilization gene

**Fig. 4:**
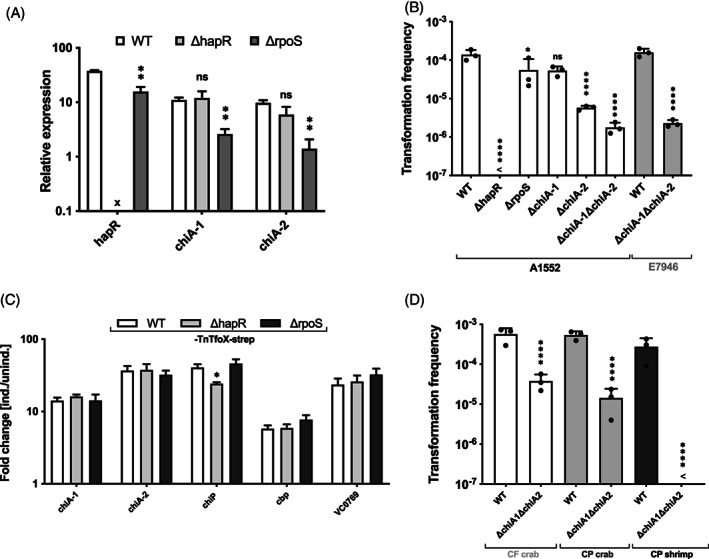
RpoS and its regulated chitinases are conditionally dispensable for transformation. A. RpoS influences chitinase transcript levels when grown on chitin. Relative expression levels of *hapR*, *chiA‐*1 and *chiA‐*2 for chitin‐grown WT, ∆hapR, or ∆rpoS *V. cholerae* strains. Details as in Fig. 2B. B. Chitinases enhance natural transformation but are not essential. The indicated WT and knock‐out strains (in A1552 or E7946 background) were tested for their natural transformability on chitin flakes (as in Fig. 2A). C. RpoS is dispensable after TfoX induction. Expression levels of representative chitin utilization genes were tested under TfoX‐non‐inducing and TfoX‐inducing conditions (see also Supporting Information Fig. S2) and the fold change upon TfoX induction is shown on the *Y*‐axis. (D) Commercially available chitin source support transformation in a chitinases‐dependent or ‐independent manner. WT and chitinase‐negative (∆chiA‐1∆chiA‐2) *V. cholerae* cells were scored for their natural transformability after their inoculation on different chitin sources: chitin flakes (CF) from crab shells, chitin powder (CP) from crab shells, and CP from shrimp. <, below detection limit. All data are based on three independent biological experiments and depict average values (±SD). Statistical analyses were performed to compare mutants to the WT using these tests: (A, C) multiple *t* tests corrected for multiple comparisons using the Holm‐Sidak method; (B, D) one‐way ANOVA with Sidak's multiple comparisons test. The detection limit was used for statistical analysis for conditions in which no transformants were recovered. *P < 0.05; **P < 0.01; ****P < 0.0001; ns, not significant. For panel C, only the statistically significant difference is shown. x, not applicable as *hapR*‐minus strain.

While the LuxO* GOF mutation explained why previous work concluded that RpoS was required for natural competence and transformation (Meibom *et al*., 2005), the question remained of why the newly generated *rpoS* mutants were highly transformable when grown on chitin flakes, contradicting recent results (Dalia, 2016), (Fig. 3A). Previously, Dalia showed that an *rpoS* mutant was barely transformable, with transformation frequencies being reduced by more than six orders of magnitude compared with the WT when grown on insoluble chitin (Dalia, 2016). This transformation deficiency was caused by the inability of the RpoS mutant to produce the extracellular chitinases ChiA‐1 and ChiA‐2 (Dalia, 2016), both of which are known to contribute to growth on chitin and to be induced by chitin oligosaccharides (Meibom *et al*., 2004). To understand why the *rpoS* mutants were fully transformable in our study, we first checked the transcript levels of *chiA‐*1 and *chiA‐*2 upon growth of WT or the *rpoS*‐minus strain on chitin. As shown in Fig. 4A, both *chiA‐*1 and *chiA‐*2 transcript levels were indeed significantly reduced in the *rpoS* mutant. Notably, the *hapR* transcripts were also slightly reduced (2.4‐fold; Fig. 4A), which most likely reflects the reduced chitin degradation capacity and growth of the *rpoS*‐minus strain. The *rpoS* mutant not only contained lower *chiA‐1* and *chiA‐2* transcript levels but also had reduced expression levels of other chitin catabolism pathway genes, such as the chitoporin‐encoding gene *chiP*, *cbp*, as well as the uncharacterized putative chitinase‐encoding gene VC0769 (Supporting Information Fig. S2A).

Given that we confirmed reduced *chiA‐*1 and *chiA‐*2 expression/transcript levels but that the *rpoS* mutant was still transformable, we wondered whether our experimental conditions did not require chitinases for competence induction. We therefore tested a ∆chiA‐1, a ∆chiA‐2, and a ∆chiA‐1∆chiA‐2 strain for natural transformation on chitin flakes and the expression of the chitin utilization genes. As shown in Fig. 4B, we observed that a *chiA‐*1‐minus strain was transformable at levels close to the WT and maintained high expression levels of *chiP*, *cbp*, and *VC0769* (Supporting Information Fig. S2A). In contrast, the *chiA‐*2‐ and *chiA‐*1*/chiA‐*2‐deficient mutants showed reduced transcript levels of the chitin utilization genes and therefore depicted transformation rates that were reduced by approximately two orders of magnitude (Fig. 4B). This latter result was confirmed for a double *chiA* knockout in a different strain background (E7946; kindly provided by A. Dalia). These results indicate that these two primary chitinases are not essential for chitin‐induced transformation on chitin flakes but do enhance its efficiency, while the RpoS mutant apparently still produced sufficient chitinases to maintain WT transformation levels.

Finally, we tested the transcript levels of chitin utilization genes in bacteria grown to high cell density in LB medium with or without artificial TfoX induction. These experiments showed lower *chiA‐*1 and *chiA‐*2 levels for the *rpoS* mutant under TfoX‐inducing conditions (Supporting Information Fig. S2B). However, the fold induction values upon TfoX production, which were observed to be between 10 and 100 for the chitin utilization genes, did not differ between a WT or *rpoS*‐minus strain (Fig. 4C). We therefore suggest that *V. cholerae* produces low levels of chitinase under starvation conditions and in the absence of carbon catabolite repression (Blokesch, 2012b). Once chitin degradation products are released from the polysaccharide and sensed by *V. cholerae*, TfoX production is triggered, making RpoS dispensable for the high‐level expression of *chiA‐*1 and *chiA‐*2 as well as other chitin utilization genes. Notably, except for *chiP*, none of the tested chitin utilization genes showed highly significant expression variations in a *hapR*‐minus strain under chitin‐grown or TfoX‐inducing conditions (Supporting Information Fig. S2). Therefore, the previous claim of a QS‐dependent regulation of chitin utilization genes is not supported (Sun *et al*., 2015).

### Different chitin sources lead to experimental variations

In contrast to a previous study (Dalia, 2016), the *rpoS*‐minus strain and the chitinase double mutant were still highly transformable when *V. cholerae* is grown on chitin. Therefore, we next tested if the chitin source could influence experimental outcomes. To our surprise, we found that the commercially available chitin flakes (#C9213) and chitin powder (#C7170) from Sigma had changed composition while maintaining the same product numbers within the past years (i.e. initially isolated from crab shells but now isolated from shrimp). While the chitin itself should not differ between these two sources, the preparation procedure might not be the same (no information was provided by the manufacturer). We therefore tested the crab‐derived chitin flakes and powder, following the standard chitin induction protocol in our laboratory (Marvig and Blokesch, 2010) with minor modifications (see Methods section), and compared those chitin sources to the new chitin powder derived from shrimp. As shown in Fig. 4D, chitin flakes and chitin powder derived from crab shells resulted in high levels of transformation of *V. cholerae* WT cells and reduced but still relatively high‐level transformation of the *chiA‐*1*/chiA‐*2 mutant. When shrimp‐derived chitin was used as sole carbon source, the WT maintained full transformability, whereas no transformants were detectable for the double mutant strain (Fig. 4D). We therefore suggest that either the input material or different preparation methods used by Sigma for crab‐ or shrimp‐derived chitin result in varying chitin mixtures, which most likely contain different ratios of the insoluble polysaccharide chitin and soluble, short‐length, chitin‐derived oligosaccharide. As the latter do not require chitinase activity for competence induction (Dalia, 2016), this could explain the different transformation outcomes.

### Natural competence and transformation are strongly conserved in seventh pandemic *V. cholerae* isolates

**Fig. 5:**
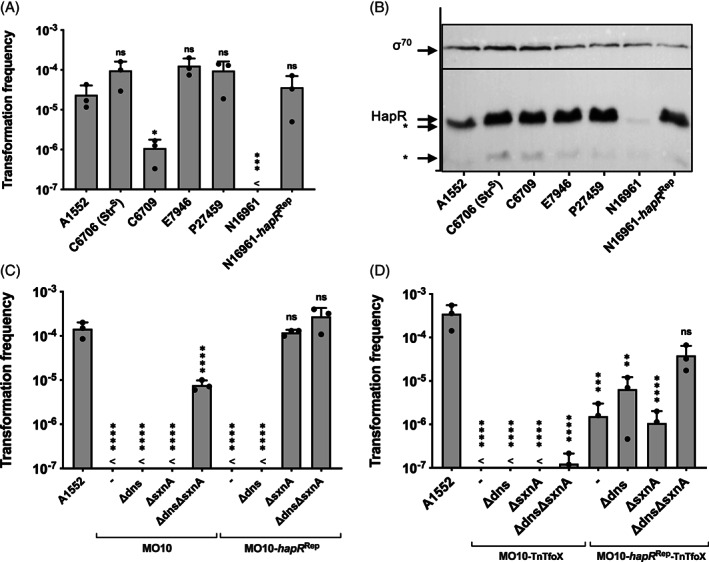
Pandemic *V. cholerae* strains are transformation variable. A, B. Different *V. cholerae* O1 El Tor pandemic isolates were tested for their natural transformability (A) or their HapR production (B). Strain N16961‐*hapR* ^Rep^ is a genetically engineered N16961 variant with a repaired *hapR* gene. C, D. The O139 serogroup strain MO10 and its *hapR*‐repaired variant (MO10‐*hapR* ^Rep^) were tested for their natural transformability on chitin (C; together with the indicated knock‐out mutants of these two strain backgrounds) or under TfoX‐inducible conditions (D). Details as described in Fig. 2. Statistical analyses were performed to compare diverse strains to the reference strain of this study, A1552, using a one‐way ANOVA on log‐transformed data with Sidak's multiple comparisons test. <, below detection limit. The detection limit was used for statistical analysis for conditions in which no transformants were recovered. *P < 0.05; **P < 0.01; ***P < 0.001; ****P < 0.0001; ns, not significant.

While we established above that experimental conditions could explain different results between research groups (e.g. RpoS‐dependent transformation phenotype), we still wanted to address whether the well‐studied seventh pandemic strains had comparable natural transformation capabilities, given that some studies reported frequencies that were more than 100‐fold above those commonly observed in our group. We therefore tested six well‐studied O1 El Tor strains for chitin‐induced natural transformation: three isolates from the Latin American cholera outbreak in Peru in the 1990s (strains A1552, C6706, and C6709), two Bangladeshi isolates from the 1970s (N16961 and P27459), and strain E7946, which was isolated in Bahrain in 1978 (Supporting Information Table S1). It should be noted that several laboratories are currently working with a QS‐impaired C6706 variant that carries a luxO* GOF mutation (luxO[G333S]; Stutzmann and Blokesch, 2016). We received a copy of this mutated C6706 variant from several laboratories, and it was transformable at levels that were one to two orders of magnitude below the level of strain A1552 (Stutzmann and Blokesch, 2016). Here, to confirm the causality of this luxO[G333S] GOF mutation for the impaired transformability, we introduced this single point mutation into strain A1552 (Supporting Information Figs. S3A and B). Given the widespread usage of the mutated C6706 variant, we decided to include an old stock of the C6706 isolate in our study (kindly provided by J. Mekalanos), which reflects a less laboratory‐domesticated version of the strain before it was rendered streptomycin resistant [named C6706 (Str^S^)]. As shown in Fig. 5, none of these other O1 El Tor pandemic strains showed lower transformation frequencies under chitin‐ (Fig. 5A) or *tfoX*‐ (Supporting Information Fig. S3C) inducing conditions when compared with the WT strain used in our laboratory (strain A1552), except for C6709 (see below) and N16961. The non‐transformability of strain N16961 was consistent with a previous report and was caused by a frameshift mutation in *hapR* that caused its QS deficiency (Meibom *et al*., 2005). Hence, repairing the single‐base‐pair mutation (strain N16961‐*hapR* ^Rep^) was sufficient to fully restore HapR production (Fig. 5B) and therefore the strain's natural transformability (Fig. 5A).

### The O139 serogroup strain MO10 is non‐transformable due to multiple factors

It has long been known that *hapR*‐mutated strains are impaired in their natural transformability, which is also true for the well‐studied strain MO10 (Blokesch and Schoolnik, 2007). This O139 serogroup strain is closely related to the O1 El Tor pandemic lineage and had acquired a novel O‐antigen‐encoding gene cluster by horizontal gene transfer (Johnson *et al*., 1994; Blokesch and Schoolnik, 2007). We therefore repaired the *hapR* copy of strain MO10 (known to carry a deleterious mutation; Joelsson *et al*., 2006) resulting in strain MO10‐*hapR* ^Rep^. Surprisingly, this strain maintained its transformation‐negative phenotype upon chitin induction (Fig. 5C) or artificial TfoX production (Fig. 5D), despite the fact that selected transcript scoring suggested a full restoration of HapR's regulatory capacity (such as *hapA* induction or *vpsA* repression; Supporting Information Fig. S4). Furthermore, the nuclease gene *dns* was repressed by the repaired HapR protein (Supporting Information Fig. S4), consistent with the fact that not even deleting *dns* restored transformation (Fig. 5C); this is in direct contrast to a transformation rescue in strain N16961 through *dns* deletion (Blokesch and Schoolnik, 2008).

We previously reported that the ATPase PilT, which is involved in DNA uptake and therefore transformation via its role in retracting the DNA‐uptake pilus, was mutated in strain MO10 (*pilT*[R206S]; Adams *et al*., 2019b). However, we excluded that this was the primary reason for the strains' non‐transformability due to the action of the secondary retraction ATPase PilU. Indeed, we showed that PilU compensates for PilT[R206S]'s impaired functionality when reconstituted in strain A1552 (Adams *et al*., 2019b). Hence, we considered that other genes related to competence or quorum sensing might be mutated in this strain and therefore whole‐genome sequenced our laboratory stock of strain MO10 and *de novo* assembled its genome (Supporting Information Table S2). Upon closer inspection of its genome, we realized that apart from the mutations in *hapR* and *pilT*, all of the genes encoding other competence‐related proteins were 100% identical to their homologues in A1552 (Supporting Information Table S3).

Intriguingly, Dalia and colleagues had previously shown that clinical *V. cholerae* isolates from Haiti were poorly transformable due to a Dns homologous nuclease IdeA, whose gene was not repressed by HapR and QstR upon competence induction (Dalia *et al*., 2015), in contrast to *dns* (Blokesch and Schoolnik, 2008). Moreover, the *ideA* gene was located on a specific integrative and conjugative element (ICE) known as the VchInd5 (Dalia *et al*., 2015). The first ICE of any *V. cholerae* strains was described by Waldor and colleagues in 1996 in strain MO10 and named SXT (Waldor *et al*., 1996). When we inspected the SXT‐contained genes for putative nuclease‐encoding genes, this search yielded locus tag H6M50_12490 in the large chromosome 1 of MO10. The gene product of H6M50_12490, which equals ORF s62 in the original SXT description (though predicted to start 33 bp downstream of the annotated start of s62; Genbank accession number AY055428.1; Beaber *et al*., 2002) shows 93%/97% and 51%/64% identity/similarity to IdeA and Dns, respectively, strongly suggesting its role as yet another extracellular nuclease (Blokesch, 2017). We therefore named this gene *sxnA* for SXT‐encoded nuclease A, and deleted the gene from the wild‐type MO10 strain; its *dns*‐minus mutant; MO10‐*hapR* ^Rep^; or MO10‐*hapR* ^Rep^∆dns. By deleting *sxnA,* the last three strains became naturally transformable on chitin surfaces, with the HapR‐repaired variants reaching frequencies in the range of those observed for strain A1552 (Fig. 5C). Similar results were obtained under chitin‐independent transformation conditions, where we observed increasing transformation frequencies for strains lacking both nuclease in the presence of a repaired *hapR* allele (Fig. 5D); the latter condition significantly induced *qstR* and *comEA* while repressing *dns* upon artificial TfoX induction (Supporting Information Fig. S4). On the basis of these results, we can conclude that the O139 serogroup strain carries, in principle, a functional competence induction system. However, competence induction is non‐functional in this strain due its QS impairment. In addition, through the horizontal acquisition of the SXT element, the strain became non‐transformable, as surrounding DNA can be degraded by the SxnA nuclease.

### Comparative genomics of *de‐novo‐*assembled pandemic *V. cholerae* genomes

**Fig. 6:**
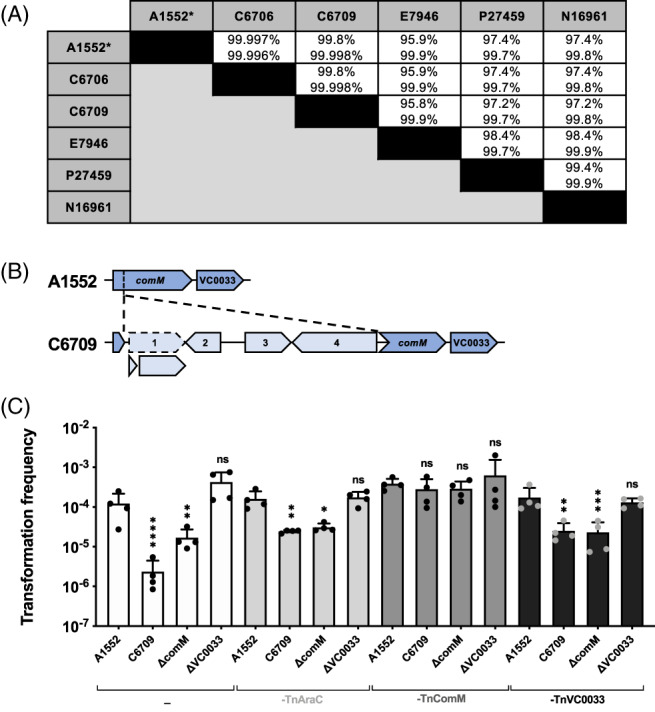
Whole genome comparisons revealed a *comM* interruption in strain C6709. A. Pairwise identity values for genome comparisons of the indicated strains (upper and lower value for chr1 and chr2, respectively). *, the intrachromosomal inversion of strain A1552 was *in silico* reverted before comparison. B. A mobile genetic elements interrupts *comM* in strain C6709. Scheme of the genomic region of *comM* in strain A1552 (dark blue) and its interruption by an MGE (light blue) in C6709. The NCBI Prokaryotic Genome Annotation Pipeline (PGAP) predicted one ORF for the region 1 (dashed arrow), despite a frameshift‐causing mutation that results in a premature stop codon (shown by the two split ORFs; mutation confirmed by Sanger sequencing). C. The interrupted *comM* is causative for the decreased transformability of strain C6709. *V. cholerae* strains A1552, C6709, and the knockout strains A1552∆comM and A1552∆VC0033 were tested for their natural transformability on chitin (+0.2% ara). The strains were either transposon‐deficient or carried a TnAraC (control), TnComM, and TnVC0033 transposon. The experiment was performed four independent times and the mean values are shown (±SD). Statistical analyses were performed to compare each strain to A1552 within the same category (e.g., −, TnAraC, TnComM, TnVC0033) using a one‐way ANOVA on log‐transformed data with Sidak's multiple comparisons test. *P < 0.05; **P < 0.01; ***P < 0.001; ****P < 0.0001; ns, not significant.

To follow up on previous claims of major strain differences within the seventh pandemic clade for several tested phenotypes, we next decided to whole‐genome sequence the above‐mentioned pandemic strains C6706(Str^S^), C6709, E7946, and P27459 using a long‐read PacBio sequencing approach and to *de novo* assemble their respective genomes. The *de novo* assembly was important, due to previous assembly artefacts, which we know from personal communications to have caused extensive experimental failures and frustration related to recombineering approaches in diverse research groups, missing rRNA clusters in old genome data, as well as a known *bona fide* genomic inversion in strain A1552 (Val *et al*., 2016; Matthey *et al*., 2018; Kemter *et al*., 2018; Matthey *et al*., 2019). Pairwise comparisons between these strains' *de‐novo‐*assembled genomes and those that we recently reported for strain A1552 and N16961 (using the same sequencing approach; Matthey *et al*., 2018; Matthey *et al*., 2019), showed overall identity levels above 95% for both chromosomes (Fig. 6A). Moreover, when we scored gene products related to chitin utilization, competence regulation, DNA‐uptake and translocation, as well as competence‐related recombination, we saw that almost all of these proteins were 100% identical (Supporting Information Table S3). From this analysis, we conclude that pandemic patient isolates are indeed highly similar, supporting previous large‐scale comparative genomic studies based on the strains' core genomes (Mutreja *et al*., 2011; Domman *et al*., 2017; Weill *et al*., 2017; Weill *et al*., 2019). Importantly for this study, all six pandemic isolates maintained their chitin‐utilization genes and their chitin‐dependent competence inducibility, suggesting that under certain circumstances, these phenotypes might be positively selected.

### Transformation is impaired in strain C6709 due to an interruption of *comM*

While the data above supported the notion that the underlying causes of non‐transformability of selected patient isolates of *V. cholerae* are often a result of an impaired QS circuit (potentially caused by sampling biases (Blokesch, 2012c), sample storage, or laboratory domestication, we wondered why transformation of strain C6709 was reduced compared to other isolates (Fig. 5A). Since we recently showed that the TfoX‐induced T6SS, which is exquisitely co‐regulated with competence induction in pandemic *V. cholerae* (Borgeaud *et al*., 2015), was fully functional in strain C6709 (Metzger *et al*., 2019), we excluded a regulatory defect. Instead, the comparative genomic analysis revealed that the *comM* competence gene in strain C6709 is interrupted by a 5′161 bp mobile genetic element (MGE) (Fig. 6B and Supporting Information Table S3). This MGE interrupted *comM* after base pair 197, causing an aborted gene product of 79 amino acids. The MGE carried four putative genes. The first gene (locus tag GTF73_00105) encoded an acyltransferase‐encoding gene, which was, however, separated into two ORFs due to a frameshift mutation. We confirmed the authenticity of this frameshift mutation by Sanger sequencing. The second gene coded for a protein of unknown function, while the third and fourth genes encoded a plasmid‐recombination enzyme and an unknown protein belonging to the recombinase family respectively.

As we and others have shown that *comM*‐deficient *V. cholerae* mutants showed reduced transformation levels (~5‐ to 10‐fold in Jaskólska *et al*., 2018 and >100‐fold in Nero *et al*., 2018; note the different transforming material used in both studies), we reasoned that *comM*'s interruption or a potential polar effect of the integrated MGE onto the *comM*‐successive gene VC0033 might be causing the lowered transformability of C6709. We therefore constructed inducible copies of *comM* and *VC0033* inside a site‐specific and *araC*‐ plus P_BAD_‐carrying transposon. We transferred those constructs (as well as the parental transposon as a control) into the chromosomes of A1552, C6709, as well as ∆comM‐ and ∆VC0033 knockout strains. We then tested the strains' natural transformability on chitin (under arabinose‐induced conditions). As shown in Fig. 6C, expression of a full‐length *comM* gene restored the transformability of strain C6709 while provision of VC0033 had no effect. The latter result is consistent with the VC0033‐minus mutant that has no transformation defect (Fig. 6C and Jaskólska et al., 2018). We also observed that even the provision of the *araC*‐carrying transposon without any inducible gene increased the transformation frequency of strain C6709 to the level of the ∆comM mutant of strain A1552. This result was highly reproducible (even in an added fourth biologically independent replicate; Fig. 6C), suggesting a positive impact of either the transposon or the AraC repressor on transformation in this strain. Based on these *comM* complementation data, we can conclude that the impairment of natural transformation in strain C6709 was indeed caused by the *comM* interruption. In support of this assumption, previous work has shown that ComM and its homolog RadA in *S. pneumoniae* (Marie *et al*., 2017; Nero *et al*., 2018) act as helicases/branch migration factors and assist in the recombination of incoming ssDNA into the double‐stranded bacterial genome through their interaction with RecA. The inactivation of *comM* in the Latin American isolate C6709, which otherwise shows more than 99.8% pairwise identity with the two other Peruvian isolates C6706 and A1552, suggests a recent acquisition of the MGE before or after sampling. Interestingly, Croucher and colleagues reported that *comM* is frequently interrupted by MGEs in other naturally transformable bacteria such as *Aggregatibacter actinomycetemcomitans*, *Acinetobacter baumannii*, and *Mannheimia succiniciproducens* (Croucher *et al*., 2016). By searching for orthologs of the apparently site‐specific integrase within the integrated MGEs, these authors identified several additional examples of *comM* interruption across diverse bacterial species including pathogens such as *Mannheimia haemolytica*, *Francisella philomiragia*, and *Pseudomonas syringae* (Croucher *et al*., 2016). Based on this finding and the assumption that *comM* interruption would abrogate transformation similarly to interruption or mutation of other core competence genes, the authors concluded that selfish MGEs tend to interrupt competence genes to limit their curing from the genome (Croucher *et al*., 2016). Natural transformation might therefore serve as a general genome maintenance tool in competent bacteria (Croucher *et al*., 2016). This is a very interesting hypothesis, and our data of the *comM* interruption in strain C6709 support the idea in principle. However, in contrast to interruptions of the essential DNA uptake/translocation genes *comEA*, *comEC*, and *comF* (Seitz and Blokesch, 2013), which are all co‐regulated with *comM* (Jaskólska *et al*., 2018), the abrogation of ComM production had only minor effects on *V. cholerae*'s transformability under the tested conditions (Fig. 6C). In our experiments, the transforming DNA was fully homologous to the competent acceptor strain A1552 and the closely related C6709 strain apart from a resistance marker, consistent with the idea that DNA is acquired from closely related, non‐kin neighbour strains through type VI secretion‐mediated predation (Borgeaud *et al*., 2015). However, ComM might contribute to a more efficient insertion of heterologous incoming DNA, as previously suggested (Nero *et al*., 2018), and interruption of its gene might indeed protect the integrated MGE from its excision. However, further mechanistic studies are needed to fully address this idea.

## Conclusions

The current study provides in‐depth insight into the conservation of chitin utilization and natural competence in the seventh pandemic clade of *V. cholerae*. This finding therefore raises new questions on the evolution of pandemic strains. For instance, *V. cholerae* O139 isolates such as strain MO10 are mostly considered as serogroup‐converted O1 El Tor strains. Such serogroup conversion events have occurred frequently in nature in the past (Chun *et al*., 2009) and can easily be accomplished by natural transformation, as we have experimentally demonstrated (Blokesch and Schoolnik, 2007). However, given the frequent possession of SXT/R391‐type ICE elements in recent isolates such as those isolated from Yemen (Weill *et al*., 2019), it is tempting to speculate that the transfer of large genomic regions such as the one that encodes the O‐antigen biosynthesis might be significantly impaired through ICE‐encoded nucleases, as previously shown for IdeA (Dalia *et al*., 2015).

We also deciphered strain‐specific competence deficiencies and showed that they are frequently based on laboratory domestication events leading to QS impairment. Moreover, we tested different chitin sources from the same commercial supplier and found that the source from which the chitin is derived or the preparation method could impact experimental outcomes. Finally, we showed that *rpoS*‐deficient *V. cholerae* are transformable in the presence of certain chitin sources and that these mutants are not impaired in QS‐related competence regulation, consistent with a previous report (Dalia, 2016). We extended this analysis to show that RpoS does not influence HapR protein production, even under chitin‐independent conditions, which supports the notion that previous connections between RpoS and HapR should be reconsidered. Furthermore, while the domestication effect in *luxO* in the *rpoS*‐deficient strain cannot be tracked back to its origin, the absence of HapR in this strain could potentially explain other observations reported in the literature (e.g., the absence of its fully complementability; Yildiz and Schoolnik, 1998). This also challenges the role of RpoS in controlling the mucosal escape response of *V. cholerae* (Nielsen *et al*., 2006) instead of mirroring the QS impairment. In light of this finding, we suggest that a proper re‐evaluation of previously described RpoS‐dependent phenotypes would provide some clarification about its role.

## Experimental procedures

### Bacterial strains, plasmids and culture conditions

Bacterial strains (*V. cholerae* and *E. coli*) and plasmids used in this study are listed in the Supporting Information Table S1. The bacteria were cultured at 30°C in Lysogeny broth medium (LB; 10 g l^−1^ of tryptone, 5 g l^−1^ of yeast extract, 10 g l^−1^ of sodium chloride; Carl Roth) medium under aerobic conditions. LB agar plates contained 1.5% w/v agar and were supplemented with antibiotics where required. The final concentrations of antibiotics were 75, 100, 100, 2.5, and 50 μg ml^−1^ for kanamycin, streptomycin, ampicillin, chloramphenicol, and gentamicin, respectively. Thiosulfate citrate bile salts sucrose (TCBS) agar was prepared following the manufacturer's instructions (Fluka). Half‐concentrated and HEPES‐buffered defined artificial seawater medium (DASW) (Meibom *et al*., 2005) was used for the natural transformation experiments on chitinous surfaces.

### *Vibrio cholerae* strain constructions

*Vibrio cholerae* mutant strains were genetically engineered using chitin‐induced transformation methods combined with flip recombination (TransFLP; Marvig and Blokesch, 2010; De Souza Silva and Blokesch, 2010; Blokesch, 2012a; Borgeaud and Blokesch, 2013). Briefly, to construct the *rpoS* and *vqmA* knockout strains, the genes' flanking regions as well as the *aph* (Kan^R^) resistance marker were PCR amplified (with Pwo polymerase; Roche) and joined by splicing using overlap extension PCR. The PCR fragments were provided to chitin‐induced competent *V. cholerae*, and natural transformants were selected using selective agar plates. Transformants were confirmed for the correctness by colony PCR (with GoTaq polymerase; Promega) followed by Sanger sequencing of the genetically engineered loci (Microsynth, Switzerland). The resistance marker was subsequently removed by flip recombination (De Souza Silva and Blokesch, 2010; Blokesch, 2012a).

The TnAraC (Adams *et al*., 2019a) miniTn7 transposon was cloned by synthesizing the fragment SYN‐araC‐NotI (IDT via LubioScience, Switzerland) containing *araC*, P_BAD_, and a *Nde*I restriction site at an optimal distance from the promoter. The synthesized fragment was PCR‐amplified, *Not*I‐digested, and cloned into an equally digested miniTn7 carried on plasmid pGP704 (Müller *et al*., 2007). To render them arabinose‐inducible, the genes *comM* and *VC0033* were subsequently cloned inside the *Nde*I cloning site of TnAraC and the resulting miniTn7 derivatives (TnComM and TnVC0033) were transferred into the respective *V. cholerae* strains by triparental mating (Bao *et al*., 1991).

### Natural transformation on chitin surfaces

To measure the natural transformation efficiency, *V. cholerae* bacteria were grown on chitin flakes as previously described with minor modifications (De Souza Silva and Blokesch, 2010; Marvig and Blokesch, 2010). Briefly, overnight cultures were back‐diluted 1:20 in 0.5x DASW + HEPES + vitamins and added to pre‐autoclaved chitin flakes (crab‐shell‐derived; #C9213, Sigma) in Eppendorf tubes. After incubation at 30°C for ~24 h, genomic DNA (gDNA) from strain A1552‐LacZ‐Kan (Marvig and Blokesch, 2010) was added as the transforming material and the mixture was incubated for 6 h. Transformants were identified by selective plating, and transformation frequencies were determined by dividing the number of resistant transformants by the total number of colony‐forming units (CFU) on plain LB agar plates. Averages of at least three biologically independent experiments are provided. Transformation frequencies were log‐transformed (Keene, 1995) and subjected to statistical analysis as indicated in the figure legends. When no transformants were recovered, the value was set to the detection limit to allow for statistical analysis (using the Prism software from GraphPad; version 8.4.3).

To test the transformation with different chitin sources (chitin flakes; crab‐shell‐ or shrimp‐shell‐derived; #C9213, Sigma; or chitin powder; #C7170, Sigma), the protocol was slightly changed. Briefly, bacteria from overnight cultures were harvested by centrifugation and resuspended in 0.5x DASW + HEPES + vitamins medium to reach an optical density at 600 nm of 0.1. An amount of 1 ml of this suspension was added to each chitin source and incubated at 30°C for ~24 h. The next day, the supernatant was replaced by fresh medium before the transforming gDNA of strain A1552‐lacZ‐Cat (Supporting Information Table S1) was added. The bacteria were detached by vortexing after another 8 h of incubation and spotted on selective and plain LB agar plates after serial dilution.

### Natural transformation in the absence of chitin

Natural transformation assays of inducible *tfoX*‐carrying strains were performed following a previously established protocol (Lo Scrudato and Blokesch, 2012; Metzger and Blokesch, 2016). Briefly, *V. cholerae* strains were grown at 30°C for 3 h in LB medium (with ot with arabinose) to an optical density at 600 nm (OD_600_) of ~1.0. At this point, 0.5 ml of the cultures were transferred to an Eppendorf tube and supplemented with 1 μg of A1552‐lacZ‐Kan gDNA. Cells were further incubated for 4 h under shaking conditions. Serial dilutions were spotted on selective and plain LB agar plates to enumerate the number of transformants and the number of total cells, respectively. Transformation frequencies were calculated as described above.

### Gene expression profiling by quantitative reverse transcription PCR

Quantitative reverse transcription PCR (qRT‐PCR)‐based transcript scoring was done for cultures grown at 30°C in LB medium for 6 h if not indicated otherwise. For chitin‐dependent expression analysis, the bacteria were inoculated onto chitin flakes submerged in 0.5x DASW + HEPES + vitamins, as noted above for the transformation assay. After an incubation period at 30°C for 24 h, the samples (four parallel tubes of each) were centrifuged for 3 min at maximum speed in a tabletop centrifuge before the supernatant was discarded. The bacteria‐ and chitin‐flakes‐containing pellet was resuspended in 1 ml of TRI reagent (Sigma) and vortexed. The chitin material was sedimented by a quick spin for 30 s at maximum speed. The supernatants was transferred to a new sterile Eppendorf tube and flash frozen on dry ice before their storage at −80°C. RNA extraction, DNase treatment, reverse transcription, and qPCR were performed as previously described (Lo Scrudato and Blokesch, 2012). Expression is shown relative to the housekeeping gene *gyrA*. Averages of at least three biologically independent experiments (±standard deviation) are provided. Statistical analyses using GraphPad's Prism (version 8.4.3) were performed on the log‐transformed data (Keene, 1995), with details provided in the figure legends.

### SDS‐PAGE and western blotting

Cell lysates were prepared as described previously (Metzger *et al*., 2016). Briefly, after growing the bacterial cultures in LB medium at 30°C for 6 h, bacterial cell pellets were resuspended in Laemmli buffer, adjusting for the total number of bacteria according to the OD_600_ values. Proteins were separated on sodium dodecyl sulfate (SDS)‐polyacrylamide gels and transferred onto PVDF membranes by western blotting. Primary antibodies against HapR (#A000542; Lo Scrudato and Blokesch, 2012) were used at 1:3000–1:5000 dilutions and goat anti‐rabbit horseradish peroxidase (HRP) (diluted 1:10 000–1:20 000; Sigma‐Aldrich, Switzerland) was used as the secondary antibody. Sigma70 detection served as a loading control using anti‐*E. coli* Sigma70 coupled with HRP (BioLegend, USA distributed via Brunschwig, Switzerland; used at a 1:10 000 dilution). Lumi‐Light^PLUS^ western blotting substrate (Roche, Switzerland) was used as an HRP substrate, and the signals were detected using a ChemiDoc XRS+ station (BioRad).

### Long‐read whole‐genome sequencing (PacBio)

Genomic DNA was isolated from the pandemic *V. cholerae* O1 El Tor strains C6706, C6709, E7946, and P27459 cultured in LB medium using Qiagen's Genomic DNA Buffer set combined with 100/G Genomic‐tips. Sequencing was performed by the Genomic Technology Facility of the University of Lausanne. For sample preparation, the DNA samples were sheared in Covaris g‐TUBEs to obtain fragments with a mean length of 20 kb.

The sheared DNA was used to prepare each library with the PacBio SMRTbell Template Prep Kit 1 according to the manufacturer's recommendations (Pacific Biosciences). The resulting libraries were size‐selected on a BluePippin system (Sage Science) for molecules larger than 15 kb. Each library was sequenced on one SMRT cell with P6/C4 chemistry and MagBeads on a PacBio RSII system at 360 min movie length. Genome assembly was performed using the protocol ‘RS_HGAP_Assembly.3' in SMRT Pipe v2.3.0, and circularization of the genomes was achieved with the Minimus assembler of the AMOS software package 3.1.0 using the default parameters (Sommer *et al*., 2007). The assembled genomes were annotated using Prokka‐1.12 (Seemann, 2014) but were re‐annotated upon submission to the NCBI database using with their own pipeline (PGAP annotation). The genomic data and NCBI accession numbers are summarized in the Supporting Information Table S2.

For the whole‐genome sequencing of strain MO10, slight variations from this protocol were applied. Briefly, the isolated DNA was sheared with a Megaruptor (Diagenode, Denville, NJ) to obtain 10–15 kb fragments on average and the size distribution was verified on a Fragment Analyser (Advanced Analytical Technologies, Ames, IA). Library preparation was done with the PacBio SMRTbell Express Template Prep Kit 2.0 (Pacific Biosciences, Menlo Park, CA, USA) according to the manufacturer's recommendations. The library was pooled with other libraries processed at the same time and the pool was size‐selected with Ampure PacBio beads to eliminate fragments <3 kb. It was sequenced with v3.0/v3.0 chemistry and diffusion loading on a PacBio Sequel instrument (Pacific Biosciences, Menlo Park, CA, USA) at 600 min movie length, pre‐extension time of 120 min using one SMRT cell 1 M v3. Genome assembly was performed using the protocol Microbial Assembly in SMRT Link Version 8.

Pairwise comparisons between the *de‐novo*‐assembled genomes were performed using the Geneious software (version 10.2.6) and a progressive Mauve algorithm assuming collinear genomes and performing full alignments. For these analyses, the large inversion between the two rRNA clusters of strain A1552 (Matthey *et al*., 2018; Matthey *et al*., 2019) was *in silico* reverted for a simplified alignment of all genomes.

While comparing the assembled genomes, we observed a frameshift mutation in the gene VC0860 (part of the minor pilin gene cluster; Seitz and Blokesch, 2013) for strain E7946. Compared to the sequence of the homologous gene in N16961 (Heidelberg *et al*., 2000) and A1552 (Matthey *et al*., 2018), the E7946 genome missed an ‘A’ base from a stretch of six consecutive As. Since we suspected that this might represent a sequencing artefact, the corresponding region was PCR amplified and Sanger sequenced. The data showed that the strain indeed carries all 6 A bases without the frameshift mutation (see also Supporting Information Table S3).

## Data Availability Statement

All sequencing data have been deposited on NCBI under BioProject accession number PRJNA598947 (BioSamples SAMN13734987, SAMN13734988, SAMN13734999, SAMN13734989, and SAMN15756141). The whole genome sequences are available in GenBank under accession numbers CP047295/CP047296 (C6706), CP047297/CP047298 (C6709), CP047303/CP047304 (E7946), CP047299/CP047300 (P27459), and CP060094/CP060095 (MO10). The raw reads are available from the Sequence Read Archive (SRA) under submission numbers SRR10828860, SRR10828861, SRR10828863, SRR10828864, and SRR12407298).

## Supporting information


**Appendix S1:** Supplementary Information
