# Supplementary material for: Comparison of chitin‐induced natural transformation in pandemic Vibrio cholerae O1 El Tor strains
Source: Environ Microbiol. 2020 Sep 9;22(10):4149–66. doi: 10.1111/1462-2920.15214 (PMC7693049; doi:10.1111/1462-2920.15214)
Supplement: Supplementary file 1 — Appendix S1: Supplementary Information [file EMI-22-4149-s001.pdf]

Supporting Information

Comparison of chitin-induced natural transformation in pandemic *Vibrio cholerae* O1 El Tor strains

Sandrine Stutzmann and Melanie Blokesch

Supplementary figures

Table S1. Bacterial strains and plasmids

Table S2. Statistics on PacBio whole-genome sequencing data and assemblies

Table S3. Conservation of selected quorum sensing, chitin utilization, and competence gene products among pandemic *V. cholerae* strains

Supporting references

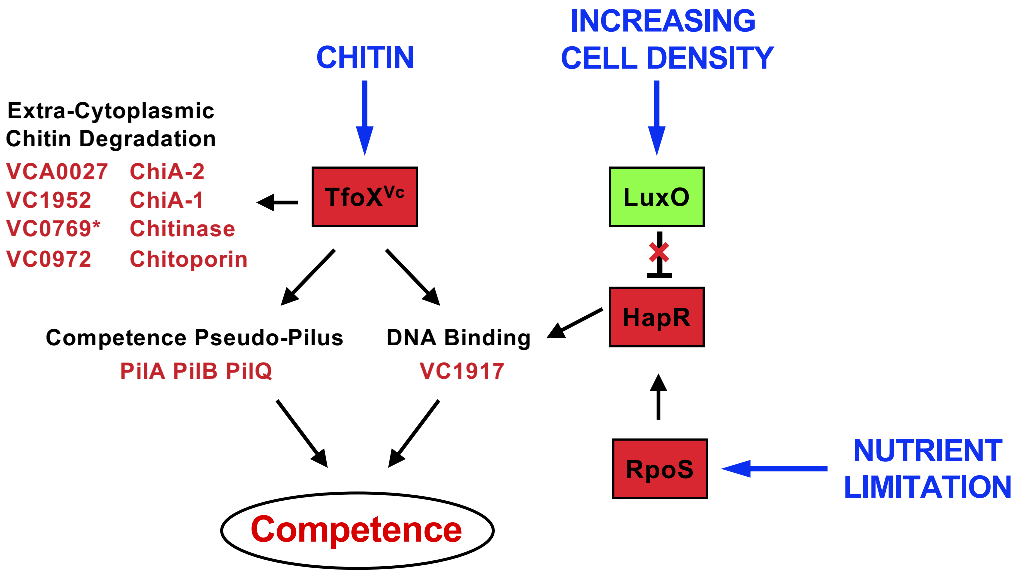

Figure S1: Competence regulatory circuit in *V. cholerae* as proposed in 2005. The model suggested three environmental variables as input: chitin, nutrient limitations, and increasing cell density. Red boxes denote positive regulators and regulated genes are shown in red writing. Freely adapted from Meibom *et al.*, 2005. *, typo in original scheme (VC0679 instead of correct locus tag/gene VC0769).

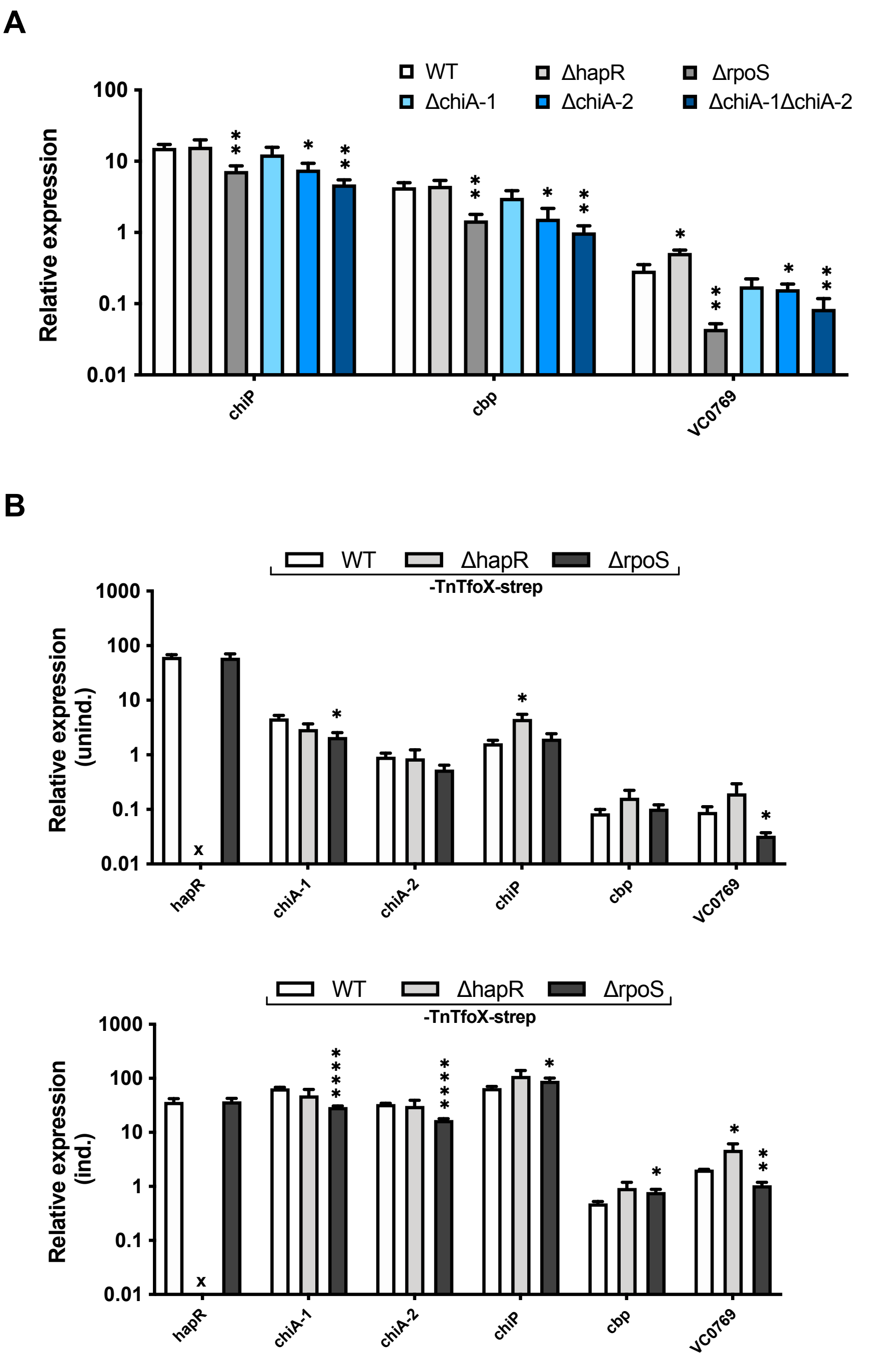

Figure S2: RpoS-dependent chitin utilization gene expression. Relative expression values for *V. cholerae* strains grown on chitin (A) or in rich medium without or with TfoX-induction (B). (A) Tested strains: WT (A1552) and its *hapR-, rpoS-, chiA-1-, chiA-2-*, and *chiA-1/chiA-2*-negative variants as indicated on top of the graph. Details as described for Fig. 4A. (B) WT, ΔhapR, and ΔrpoS strains carrying inducible *tfoX* on a transposon (TnTfoX-strep) were grown in the absence (uninduced; top graph) or presence (induced; lower graph) of the inducer arabinose (0.2% ara). Data in both panels are based on three independent biological replicates and show the average values (±SD). Statistical analyses on log-transformed data were performed to compare mutants to their WT parental strain using multiple *t* tests corrected for multiple comparisons using the Holm-Sidak method. * $p<0.05$; ** $p<0.01$; ***, $p<0.001$; ****, $p<0.0001$; ns, not significant. x, not applicable as *hapR*-minus strain.

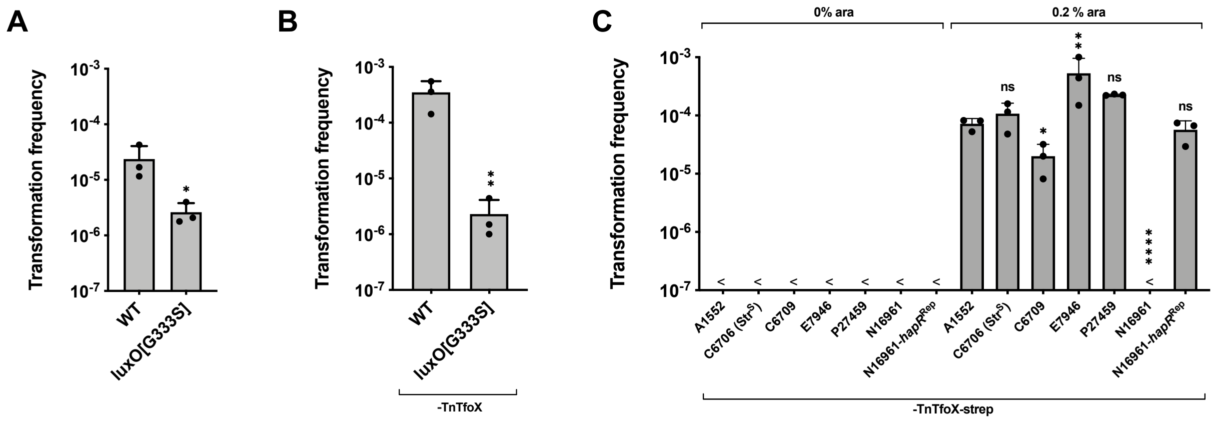

Figure S3: Transformation defects in pandemic O1 *V. cholerae* strains. Transformation frequencies were scored under (A) chitin- or (B and C) TfoX-dependent conditions. Details as in Fig. 2A and Fig. 3C for panels (A) and (B and C), respectively. For panel B, the two strains were grown in the presence of 0.02% ara. Data are based on three independent experiments and the bars show the average values (±SD). <, below detection limit. Statistical analyses were performed to compare the mutant or the different pandemic isolates to strain A1552 (in each category for panel C; e.g., 0% or 0.2% ara) using these tests: (A and B) Unpaired *t*-test; (C) one-way ANOVA with Sidak's multiple comparisons test. * p<0.05; ** p<0.01; ****, p<0.0001; ns, not significant.

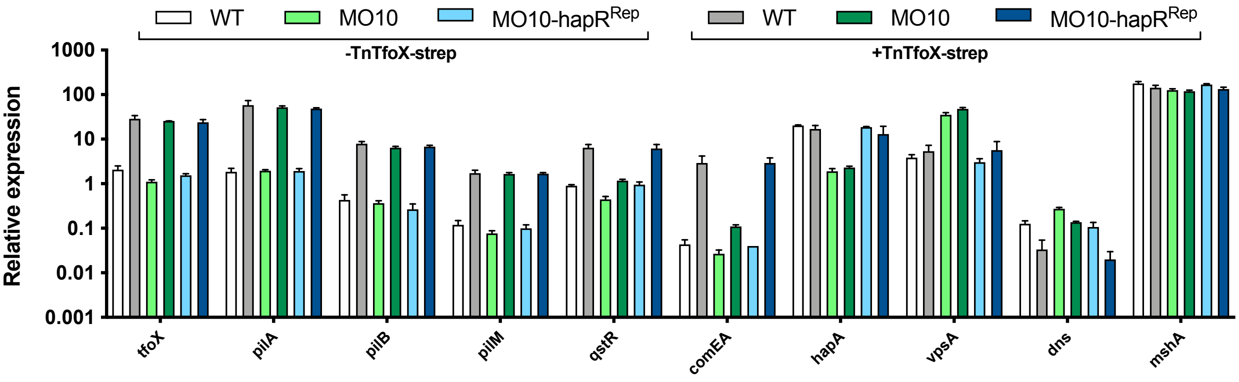

Figure S4: Impaired competence gene expression in strain MO10. The O1 strain A1552 and the O139 serogroup strain MO10 and its *hapR*-restored variant (MO10-*hapR*^{Rep}) were grown in the presence of 0.02% ara. The strains carried no transposon (-TnTfoX-strep) or the tfoX-carrying transposon (+TnTfoX-strep). Transcript levels were scored by qRT-PCR. Relative expression levels are averages from three independent experiments (±SD) and shown on the *Y*-axis. The *mshA* gene served as a control for HapR- and TfoX-independent expression.

Table S1. Bacterial strains and plasmids

Strains or plasmids	Genotype/description*	Internal strain number (accession #)	Reference (original strain and genome sequence)
V. cholerae			
A1552	Wild-type; O1 El Tor Inaba; Rif^R	MB#1; CP028894 CP028895 (Blokesch lab stock; Matthey *et al.*, 2018)	(Yildiz and Schoolnik, 1998)
A1552-LacZ-Kan	A1552 strain with *aph* cassette in *lacZ* gene; Rif^R, Kan^R	MB#135	(De Souza Silva and Blokesch, 2010)
A1552-LacZ-Cat	A1552 strain with *cat* cassette in *lacZ* gene; Rif^R, Cm^R	MB#4608	This study
ΔcqsA	A1552 with *cqsA* deleted (A1552ΔVCA0523); Rif^R	MB#559	(Suckow *et al.*, 2011)
ΔluxS	A1552 with *luxS* deleted (A1552ΔVC0557); Rif^R	MB#23	(Nielsen *et al.*, 2006)
ΔluxSΔcqsA	A1552 with *luxS* and *cqsA* deleted (A1552ΔVC0557ΔVCA0523); Rif^R	MB#602	(Suckow *et al.*, 2011)
ΔhapR	A1552 with *hapR* deleted (A1552ΔVC0583); Rif^R	MB#3	(Meibom *et al.*, 2005)
C6706 (C6706str)	C6706str; El Tor biotype, O1; Str^R	MB#4242	(Antonova and Hammer, 2011); Hammer laboratory strain C6706str
C6706ΔcqsA	C6706strΔcqsA; Str^R	MB#4246	(Antonova and Hammer, 2011); Hammer laboratory strain BH1523
C6706ΔluxS	C6706strΔluxS; Str^R	MB#4248	(Antonova and Hammer, 2011); Hammer laboratory strain BH1515
C6706ΔluxSΔcqsA	C6706strΔluxSΔcqsA; Str^R	MB#4244	(Antonova and Hammer, 2011); Hammer laboratory strain BH1575

C6706ΔhapR	C6706str ΔhapR; Str^R	MB#4252	(Antonova and Hammer, 2011); Hammer laboratory strain BH1543
ΔvqmA	A1552 with *vqmA* deleted using TransFlp method (A1552ΔvqmA::FRT); Rif^R	MB#3809	This study
rpoS::cm	A1552 with *cat* cassette interrupting *rpoS*; Rif^R; Cm^R	MB#7 (also #15 & #22)	Laboratory stock; transferred from Schoolnik laboratory FY1; (Yildiz and Schoolnik, 1998)
rpoS::cm-new	A1552 naturally transformed with gDNA of strain *rpoS::cm*; Rif^R, Cm^R	MB#4984	This study
ΔrpoS	A1552 with *rpoS* deleted using TransFlp method (A1552ΔrpoS::FRT); Rif^R	MB#4486	This study
ΔchiA-1	A1552 with *chiA-1* deleted (A1552ΔVC1952); Rif^R	MB#350	(Meibom *et al.*, 2004)
ΔchiA-2	A1552 with *chiA-2* deleted (A1552ΔVCA0027); Rif^R	MB#349	(Meibom *et al.*, 2004)
ΔchiA-1ΔchiA-2	A1552 with *chiA-1* and *chiA-2* deleted (A1552ΔVC1952ΔVCA0027); Rif^R	MB#351	(Meibom *et al.*, 2004)
E7946	E7946; Str^R	MB#5078	(Dalia, 2016); Dalia laboratory strain SAD030
E7946ΔchiA-1 ΔchiA-2	E7946 delta ChiA1, delta ChiA2; Str^R	MB#5081	(Dalia, 2016); Dalia laboratory strain SAD863
A1552-luxO[G333S]-	A1552 with site-directed point mutation in *luxO* (resulting in LuxO[G333S]); Rif^R	MB#4492	This study
A1552-luxO[G333S]- Tn*tfoX*	A1552-luxO[G333S] containing mini-Tn7-*araC*-P_{BAD}-*tfoX*; Rif^R, $Gent^R$	MB# 5932	This study
C6706 (Str^S)	C6706; O1 El Tor Inaba; isolated in 1991, Peru; original isolate before introduction of streptomycin resistance mutation; non-mutated *luxO*; Str^S	MB#4522 CP047295 CP047296 (Blokesch lab stock; this study)	Gift from J. Mekalanos (Harvard); (Van der Henst *et al.*, 2018)
C6709	C6709; O1 El Tor Inaba; isolated in 1991, Peru; Str^R	MB#1503 CP047297 CP047298	Laboratory stock; (Wachsmuth *et al.*, 1993)

		(Blokesch lab stock; this study)	
E7946	E7946; O1 El Tor Ogawa; isolated in 1978, Bahrain; Str^R	MB#2600 CP047303 CP047304 (Blokesch lab stock; this study)	Laboratory stock; (Miller *et al.*, 1989)
P27459	P27459; O1 El Tor Inaba; isolated in 1976, Bangladesh; Str^R	MB#1504 CP047299 CP047300 (Blokesch lab stock; this study)	Laboratory stock; (Pearson *et al.*, 1993)
N16961	N16961; O1 El Tor Inaba; isolated in 1975, Bangladesh; *hapR* frame-shift, Str^R	MB#2 CP028827 CP028828 (Blokesch lab stock; Matthey *et al.*, 2018)	Laboratory stock; (Heidelberg *et al.*, 2000))
N16961-*hapR*^{Rep}	N16961 with repaired *hapR* frameshift mutation (scarless); Str^R	MB#5663	(Van der Henst *et al.*, 2018)
A1552-Tn*tfoX-strep*	A1552 containing mini-Tn7-*araC*-P_{BAD}-*tfoX-strep*; Rif^R, $Gent^R$	MB#3420	(Metzger *et al.*, 2016)
C6706 (Str^S) - Tn*tfoX-strep*	C6706 (previously named C6706-original) containing mini-Tn7-*araC*-P_{BAD}-*tfoX-strep*; Str^S, $Gent^R$	MB#4530	This study
C6709-Tn*tfoX-strep*	C6709 containing mini-Tn7-*araC*-P_{BAD}-*tfoX-strep*; Str^R, $Gent^R$	MB#4156	This study
E7946-Tn*tfoX-strep*	E7946 containing mini-Tn7-*araC*-P_{BAD}-*tfoX-strep*; Str^R, $Gent^R$	MB#4162	This study
P27459-Tn*tfoX-strep*	P27459 containing mini-Tn7-*araC*-P_{BAD}-*tfoX-strep*; Str^R, $Gent^R$	MB#4158	This study
N16961-Tn*tfoX-strep*	N16961 containing mini-Tn7-*araC*-P_{BAD}-*tfoX-strep*; Str^R, $Gent^R$	MB#4395	This study
N16961-*hapR*^{Rep}-Tn*tfoX-strep*	N16961-*hapR*^{Rep} containing mini-Tn7-*araC*-P_{BAD}-*tfoX-strep*; Str^R, $Gent^R$	MB#5697	This study
MO10	O139 strain; isolated in 1992, India; *hapR* mutated; Str^R	MB#5 CP060094 CP060095 (Blokesch lab stock; this study)	Laboratory stock; (Waldor and Mekalanos, 1994)
MO10-*hapR*^{Rep}	MO10 with repaired *hapR* gene; Str^R	MB#5661	(Adams *et al.*, 2019b)
MO10Δdns	MO10 with *dns* deleted (ΔVC0470); Str^R	MB#472	This study

MO10ΔsxnA	MO10 with *sxnA* deleted (locus tag H6M50_12490 in sequence reported in this study or ORF s62 in AY055428.1; (Beaber *et al.*, 2002)); Str^R	MB#4868	This study
MO10ΔdnsΔsxnA	MO10 with *dns* and *sxnA* deleted; Str^R	MB#4869	This study
MO10-*hapR*^{Rep}Δdns	MO10-*hapR*^{Rep} with *dns* deleted; Str^R	MB#5767	This study
MO10-*hapR*^{Rep} ΔsxnA	MO10-*hapR*^{Rep} with *sxnA* deleted; Str^R	MB#5763	This study
MO10-*hapR*^{Rep} ΔdnsΔsxnA	MO10-*hapR*^{Rep} with *dns* and *sxnA* deleted; Str^R	MB#5799	This study
A1552-TntfoX	A1552 containing mini-Tn7-*araC*-P_{BAD}-*tfoX*; Rif^R, $Gent^R$	MB#1626	(Lo Scrudato and Blokesch, 2012)
MO10-TntfoX	MO10 containing mini-Tn7-*araC*-P_{BAD}-*tfoX*; Str^R, $Gent^R$	MB#5924	(Adams *et al.*, 2019b)
MO10-*hapR*^{Rep}-TntfoX	MO10-*hapR*^{Rep} containing mini-Tn7-*araC*-P_{BAD}-*tfoX*; Str^R, $Gent^R$	MB#5690	(Adams *et al.*, 2019b)
MO10Δdns-TntfoX	MO10Δdns containing mini-Tn7-*araC*-P_{BAD}-*tfoX*; Str^R, $Gent^R$	MB#5925	This study
MO10ΔsxnA-TntfoX	MO10ΔsxnA containing mini-Tn7-*araC*-P_{BAD}-*tfoX*; Str^R, $Gent^R$	MB#5926	This study
MO10ΔdnsΔsxnA-TntfoX	MO10ΔdnsΔsxnA containing mini-Tn7-*araC*-P_{BAD}-*tfoX*; Str^R, $Gent^R$	MB#5927	This study
MO10-*hapR*^{Rep} Δdns-TntfoX	MO10-*hapR*^{Rep}Δdns containing mini-Tn7-*araC*-P_{BAD}-*tfoX*; Str^R, $Gent^R$	MB#5768	This study
MO10-*hapR*^{Rep} ΔsxnA-TntfoX	MO10-*hapR*^{Rep}ΔsxnA containing mini-Tn7-*araC*-P_{BAD}-*tfoX*; Str^R, $Gent^R$	MB#5764	This study
MO10-*hapR*^{Rep} Δdns ΔsxnA-TntfoX	MO10-*hapR*^{Rep} ΔdnsΔsxnA containing mini-Tn7-*araC*-P_{BAD}-*tfoX*; Str^R, $Gent^R$	MB#5800	This study
ΔcomM	A1552 with *comM* deleted (VC0032); Rif^R	MB#381	(Jaskólska *et al.*, 2018)
ΔVC0033	A1552 with *VC0033* deleted using TransFlp method (A1552ΔVC0033::FRT); Rif^R	MB#6127	(Jaskólska *et al.*, 2018)
A1552-TnAraC	A1552 containing mini-Tn7-*araC* (TnAraC); Rif^R, $Gent^R$	MB#8503	This study
C6709-TnAraC	C6709 containing mini-Tn7-*araC* (TnAraC); Str^R, $Gent^R$	MB#8504	This study
ΔcomM-TnAraC	ΔcomM containing mini-Tn7-*araC* (TnAraC); Rif^R, $Gent^R$	MB#8505	This study
ΔVC0033 -TnAraC	ΔVC0033 containing mini-Tn7-*araC* (TnAraC); Rif^R, $Gent^R$	MB#8506	This study
A1552-TnComM	A1552 containing mini-Tn7-*araC-comM* (TnComM); Rif^R, $Gent^R$	MB#8507	This study
C6709-TnComM	C6709 containing mini-Tn7-*araC-comM* (TnComM); Str^R, $Gent^R$	MB#8508	This study
ΔcomM-TnComM	ΔcomM containing mini-Tn7-*araC-comM* (TnComM); Rif^R, $Gent^R$	MB#8509	This study
ΔVC0033-TnComM	ΔVC0033 containing mini-Tn7-*araC-comM* (TnComM); Rif^R, $Gent^R$	MB#8510	This study
A1552-TnVC0033	A1552 containing mini-Tn7-*araC-VC0033* (TnVC0033); Rif^R, $Gent^R$	MB#8511	This study

C6709-TnVC0033	C6709 containing mini-Tn7-*araC-VC0033* (TnVC0033); Str^R, $Gent^R$	MB#8512	This study
ΔcomM-TnVC0033	ΔcomM containing mini-Tn7-*araC-VC0033* (TnVC0033); Rif^R, $Gent^R$	MB#8513	This study
ΔVC0033-TnVC0033	ΔVC0033 containing mini-Tn7-*araC-VC0033* (TnVC0033); Rif^R, $Gent^R$	MB#8514	This study
E. coli			
TOP10	F- mcrA Δ(mrr-hsdRMS-mcrBC) φ80lacZΔM15 ΔlacX74 nupG recA1 araΔ139 Δ(ara-leu)7697 galE15 galK16 rpsL(Str^R) endA1λ^-	MB#741	Invitrogen
DH5α	F^- endA1 glnV44 thi-1 recA1 relA1 gyrA96 deoR nupG φ80lacZΔM15 Δ(lacZYA-argF) U169 hsdR17 (r_K^- m_K^+) phoA, λ-	MB#736	(Yanisch-Perron *et al.*, 1985)
SM10λpir	thi-1 thr leu tonA lacY supE recA::RP4-2-Tc::Mu, Kmr (λpir); Kan^R	MB#647	(Simon *et al.*, 1983)
S17-1λpir	Tpr Smr recA thi pro hsdR2M1 RP4:2-Tc:Mu:Kmr Tn7 (λpir); Str^R	MB#648	(Simon *et al.*, 1983)
Plasmids			
pGP704-Sac28	suicide vector, ori R6K *sacB*; Amp^R	MB#649	(Meibom *et al.*, 2004)
pSac28ΔsxnA	pSac28ΔH6M50_12490 (nuclease gene on SXT element of MO10); Amp^R	MB#4833	This study
pUX-BF-13	*ori*R6K, helper plasmid with Tn7 transposition function; Amp^R	MB#457	(Bao *et al.*, 1991)
pBR-flp	FLP^+, λ $cI857^+$, λ p_R from pCP20 integrated into *Eco*RV site of pBR322	MB#1203	(De Souza Silva and Blokesch, 2010)
pBR-FRT-Kan-FRT2	pBR322 derivative containing improved FRT-*aph*-FRT cassette; used as template for TransFLP; Amp^R, Kan^R	MB#3782	(Metzger *et al.*, 2016)
pBR-FRT-Cat-FRT2	pBR322 derivative containing improved FRT-*cat*-FRT cassette; used as template for TransFLP; AmpR, CmR	MB#3783	(Metzger *et al.*, 2016)
pGP704-mTn*tfoX*	pGP704 with mini-Tn7 carrying *araC* and P_{BAD}-driven *tfoX*; Amp^R, $Gent^R$	MB#1624	(Lo Scrudato and Blokesch, 2012)
pGP704-mTn*tfoX-strep*	pGP704 with mini-Tn7 carrying *araC* and P_{BAD}-driven *tfoX-strep*; Amp^R, $Gent^R$	MB#3664	(Metzger *et al.*, 2016)
pGP704-Tn	pGP704 with mini-Tn7; Amp^R, $Gent^R$	MB#645	(Müller *et al.*, 2007)
pGP704-TnAraC	pGP704 with mini-Tn7 carrying *araC* and P_{BAD}; Amp^R, $Gent^R$	MB#5513	This study & (Adams *et al.*, 2019a)
pGP704-TnComM	pGP704 with mini-Tn7 carrying *araC* and P_{BAD}-driven *comM*; Amp^R, $Gent^R$	MB#8500	This study
pGP704-TnVC0033	pGP704 with mini-Tn7 carrying *araC* and P_{BAD}-driven *VC0033*; Amp^R, $Gent^R$	MB#8502	This study

* Locus tag numbers (VC…./VCA….) according to (Heidelberg *et al.*, 2000)

Table S2. Statistics on PacBio whole-genome sequencing data and assemblies

	C6706	C6709	E7946	P27459	MO10
**Strain ID**	MB#4522	MB#1503	MB#2600	MB#1504	MB#5
**BioSample ID**	SAMN13734987	SAMN13734988	SAMN13734989	SAMN13734999	SAMN15756141
**GenBank accession numbers (chr 1 / chr 2)**	CP047295 / CP047296	CP047297 / CP047298	CP047303 / CP047304	CP047299 / CP047300	CP060094 / CP060095
**Number of bases**	1,908,280,912	1,966,394,792	2,120,148,521	2,112,220,841	741,322,034
**Number of reads**	107,132	113,782	118,563	118,978	117,801
**Mean read length**	17,812 bp	17,282 bp	17,882 bp	17,753 bp	34,264
**Total number of contigs**	2 (chr1+chr2)	2 (chr1+chr2)	2 (chr1+chr2)	2 (chr1+chr2)	2 (chr1; linear +chr2)
**Contig length after circularization**	3'015'077 bp (chr1)<br>1'070'354 bp (chr 2)	3,020,257 bp (chr 1)<br>1,070,357 bp (chr 2)	3,011,453 bp (chr 1)<br>1,071,008 bp (chr 2)	2,962,956 bp (chr 1)<br>1,073,557 bp (chr 2)	3,102,362 bp (chr 1)<br>1,073,557 bp (chr 2)
**Total genome size**	4,085,431 bp	4,090,614 bp	4,082,461 bp	4,036,513 bp	4,138,826 bp
**Mean coverage**	329 x	343 x	344 x	375 x	174 x
**GC content**	47.7% (chr1)<br>46.9% (chr2)	47.6% (chr1)<br>46.9% (chr2)	47.7% (chr1)<br>46.9% (chr2)	47.7% (chr1)<br>46.9% (chr2)	47.7% (chr1)<br>47.1% (chr2)

Table S3. Conservation of selected quorum sensing, chitin utilization, and competence gene products among pandemic *V. cholerae* strains

Category	VC locus tag number [(1)] (gene name)	Protein identity / similarity compared to strain A1552					
		**N16961 [(2)]**	**C6706**	**C6709**	**E7946**	**P27459**	**MO10**
**Chitin utilization & sensing**	**VC0611-VC619**	100%	100%	100%	100%	100%	100%
	**VC0620 (cbp)**	100%	100%	100%	100%	100%	100%
	**VC0622 (chiS)**	100%	100%	100%	100%	100%	100%
	**VC0972 (chiP)**	100%	100%	100%	100%	100%	100%
	**VC0994 (nagA)**	100%	100%	100%	100%	100%	100%
	**VC0995 (nagE)**	100%	100%	100%	100%	100%	100%
	**VC1073**	100%	100%	100%	100%	100%	100%
	**VC1280-86**	100%	100%	100%	100%	100%	100%
	**VC1783**	100%	100%	100%	100%	100%	100%
	**VC1952 (chiA-1)**	100%	100%	100%	100%	100%	100%
	**VCA0027 (chiA-2)**	100%	100%	100%	100%	100%	100%
	**VC2217**	100%	100%	100%	100%	100%	100%
	**VCA0140**	100%	100%	100%	100%	100%	100%
	**VCA0700 (chiD)**	100%	100%	100%	100%	100%	100%
	**VCA1025 (nagB)**	100%	100%	100%	100%	100%	100%
	**VC2080 (tfoS)**	100%	100%	100%	100%	100%	100%
	**VC0534 (rpoS)**	100%	100%	100%	100%	100%	100%
**QS & competence regulation**	**VC0583 (hapR)**	**frameshift mutation [(3)]**	100%	100%	100%	100%	**99.5% [(4)]**
	**VC1153 (tfoX)**	100%	100%	100%	100%	100%	100%
	**VC0396 (qstR)**	100%	100%	100%	100%	100%	100%
	**VC1021 (luxO)**	100%	**100% [(5)]**	100%	100%	100%	100%
	**VC2614 (crp)**	100%	100%	100%	100%	100%	100%
	**VC2677 (cytR)**	100%	100%	100%	100%	100%	100%
**DNA-uptake complex (including type IV pilus)**	**VC2423 (pilA)**	100%	100%	100%	100%	100%	100%
	**VC2424 (pilB)**	100%	100%	100%	100%	100%	100%
	**VC2425 (pilC)**	100%	100%	100%	100%	100%	100%
	**VC2426 (pilD)**	100%	100%	100%	100%	100%	100%

**VC1612 (pilF)**	**VC2630 (pilQ)**	100%	100%	100%	100%	100%	100%
	**VC2631 (pilP)**	100%	100%	100%	100%	100%	100%
	**VC2632 (pilO)**	100%	100%	100%	100%	100%	100%
	**VC2633 (pilN)**	100%	100%	100%	100%	100%	100%
	**VC2634 (pilM)**	100%	100%	100%	100%	100%	100%
	**VC1612 (pilF)**	100%	100%	100%	100%	100%	100%
	**VC0462 (pilT)**	100%	100%	100%	100%	100%	**99.7% (6)**
	**VC0463 (pilU)**	100%	100%	100%	100%	100%	100%
	**VC0857**	100%	100%	100%	100%	100%	100%
	**VC0858**	100%	100%	100%	100%	100%	100%
	**VC0859**	100%	100%	100%	100%	100%	100%
	**VC0860**	100%	100%	100%	**100% (7)**	100%	100%
	**VC0861**	100%	100%	100%	100%	100%	100%
**DNA uptake & translocation**	**VC1917 (comEA)**	100%	100%	100%	100%	100%	100%
	**VC1879 (comEC)**	100%	100%	100%	100%	100%	100%
	**VC2719 (comF)**	100%	100%	100%	100%	100%	100%
**DNA protection & recombination**	**VC0048 (dprA)**	100%	100%	100%	100%	100%	100%
	**VC0543 (recA)**	**99.7% (8)**	100%	100%	100%	100%	100%
	**VC0032 (comM)**	100%	100%	**gene interrupted (9)**	100%	100%	100%

(1) Locus tag numbers according to (Heidelberg *et al.*, 2000);
(2) N16961 initial genome sequence by Heidelberg *et al.*, 2000 (NC_002505/NC_002506) as well as re-sequenced laboratory collection strain (accession numbers CP028827/CP028828).
(3) Gene identity 99.8%; T218 missing in *hapR* of N16961 causing frameshift and shorten ORF to 241bp instead of full-length gene of 612 bp in A1552.
(4) See main text for information on *hapR* defect in strain MO10, which encodes HapR[R12L], confirming a previous study (Joelsson *et al.*, 2006).
(5) *luxO* correct in the old version of strain C6706 (still Str^S) used in this study; several laboratories work with domesticated version of C6706 that carries *luxO** GOF mutation encoding LuxO[G333S]; (Stutzmann and Blokesch, 2016).
(6) *pilT* in newly sequenced genome of MO10 strain (accession numbers CP060094/CP060095) carries mutation in *pilT* encoding PilT[R206S], as we had previously reported; (Adams *et al.*, 2019b).
(7) Frameshift mutation in new genome sequence of strain E7946 (accession numbers CP047303/ CP047304); shown to be a sequencing artifact by Sanger sequening.
(8) Strain N16961 harbors a mutation in *recA* leading the an amino acid exchange C305Y. We confirmed that this change is not based on a sequencing artifact but also present in the original N16961 sequence (Heidelberg *et al.*, 2000). It should be noted that the ORF annotation is incorrect in the original genome sequence rendering the RecA protein 58 amino acids longer at its N-terminus.
(9) See main text for information on *comM* interruption in strain C6709.

Supporting References

Adams, D.W., Stutzmann, S., Stoudmann, C., and Blokesch, M. (2019a) DNA-uptake pili of *Vibrio cholerae* are required for chitin colonization and capable of kin recognition via sequence-specific self-interaction. *Nat Microbiol* **4**: 1545-1557.

Adams, D.W., Pereira, J.M., Stoudmann, C., Stutzmann, S., and Blokesch, M. (2019b) The type IV pilus protein PilU functions as a PilT-dependent retraction ATPase. *PLoS Genet* **15**: e1008393.

Antonova, E.S., and Hammer, B.K. (2011) Quorum-sensing autoinducer molecules produced by members of a multispecies biofilm promote horizontal gene transfer to *Vibrio cholerae*. *FEMS Microbiol Lett* **322**: 68-76.

Bao, Y., Lies, D.P., Fu, H., and Roberts, G.P. (1991) An improved Tn*7*-based system for the single-copy insertion of cloned genes into chromosomes of Gram-negative bacteria. *Gene* **109**: 167-168.

Beaber, J.W., Hochhut, B., and Waldor, M.K. (2002) Genomic and functional analyses of SXT, an integrating antibiotic resistance gene transfer element derived from *Vibrio cholerae*. *J Bacteriol* **184**: 4259-4269.

Dalia, A.B. (2016) RpoS is required for natural transformation of *Vibrio cholerae* through regulation of chitinases. *Environ Microbiol* **18**: 3758-3767.

De Souza Silva, O., and Blokesch, M. (2010) Genetic manipulation of *Vibrio cholerae* by combining natural transformation with FLP recombination. *Plasmid* **64**: 186-195.

Heidelberg, J.F., Eisen, J.A., Nelson, W.C., Clayton, R.A., Gwinn, M.L., Dodson, R.J. *et al.* (2000) DNA sequence of both chromosomes of the cholera pathogen *Vibrio cholerae*. *Nature* **406**: 477-483.

Jaskólska, M., Stutzmann, S., Stoudmann, C., and Blokesch, M. (2018) QstR-dependent regulation of natural competence and type VI secretion in *Vibrio cholerae*. *Nucleic Acids Res* **46**: 10619-10634.

Joelsson, A., Liu, Z., and Zhu, J. (2006) Genetic and phenotypic diversity of quorum-sensing systems in clinical and environmental isolates of *Vibrio cholerae*. *Infect Immun* **74**: 1141-1147.

Lo Scrudato, M., and Blokesch, M. (2012) The regulatory network of natural competence and transformation of *Vibrio cholerae*. *PLoS Genet* **8**: e1002778.

Marvig, R.L., and Blokesch, M. (2010) Natural transformation of *Vibrio cholerae* as a tool-optimizing the procedure. *BMC Microbiol* **10**: 155.

Matthey, N., Drebes Dörr, N.C., and Blokesch, M. (2018) Long-Read-Based Genome Sequences of Pandemic and Environmental *Vibrio cholerae* Strains. *Microbiol Resour Announc* **7**: e01574-01518.

Meibom, K.L., Blokesch, M., Dolganov, N.A., Wu, C.-Y., and Schoolnik, G.K. (2005) Chitin induces natural competence in *Vibrio cholerae*. *Science* **310**: 1824-1827.

Meibom, K.L., Li, X.B., Nielsen, A.T., Wu, C.Y., Roseman, S., and Schoolnik, G.K. (2004) The *Vibrio cholerae* chitin utilization program. *Proc Natl Acad Sci USA* **101**: 2524-2529.

Metzger, L.C., Stutzmann, S., Scrignari, T., Van der Henst, C., Matthey, N., and Blokesch, M. (2016) Independent Regulation of Type VI Secretion in *Vibrio cholerae* by TfoX and TfoY. *Cell Rep* **15**: 951-958.

Miller, V.L., DiRita, V.J., and Mekalanos, J.J. (1989) Identification of *toxS*, a regulatory gene whose product enhances ToxR-mediated activation of the cholera toxin promoter. *J Bacteriol* **171**: 1288-1293.

Müller, J., Miller, M.C., Nielsen, A.T., Schoolnik, G.K., and Spormann, A.M. (2007) *vpsA*- and *luxO*-independent biofilms of *Vibrio cholerae*. *FEMS Microbiol Lett* **275**: 199-206.

Nielsen, A.T., Dolganov, N.A., Otto, G., Miller, M.C., Wu, C.Y., and Schoolnik, G.K. (2006) RpoS controls the *Vibrio cholerae* mucosal escape response. *PLoS Pathog* **2**: e109.

Pearson, G.D., Woods, A., Chiang, S.L., and Mekalanos, J.J. (1993) CTX genetic element encodes a site-specific recombination system and an intestinal colonization factor. *Proc Natl Acad Sci USA* **90**: 3750-3754.

Simon, R., Priefer, U., and Pühler, A. (1983) A broad host range mobilization system for *in vivo* genetic engineering: transposon mutagenesis in Gram negative bacteria. *Nat Biotechnol* **1**: 784-791.

Stutzmann, S., and Blokesch, M. (2016) Circulation of a Quorum-Sensing-Impaired Variant of *Vibrio cholerae* Strain C6706 Masks Important Phenotypes. *mSphere* **1**: e00098-16.

Suckow, G., Seitz, P., and Blokesch, M. (2011) Quorum sensing contributes to natural transformation of *Vibrio cholerae* in a species-specific manner. *J Bacteriol* **193**: 4914-4924.

Van der Henst, C., Vanhove, A.S., Drebes Dörr, N.C., Stutzmann, S., Stoudmann, C., Clerc, S. *et al.* (2018) Molecular insights into *Vibrio cholerae*'s intra-amoebal host-pathogen interactions. *Nature Commun* **9**: 3460.

Wachsmuth, I.K., Evins, G.M., Fields, P.I., Olsvik, O., Popovic, T., Bopp, C.A. *et al.* (1993) The molecular epidemiology of cholera in Latin America. *J Infect Dis* **167**: 621-626.

Waldor, M.K., and Mekalanos, J.J. (1994) ToxR regulates virulence gene expression in non-O1 strains of *Vibrio cholerae* that cause epidemic cholera. *Infect Immun* **62**: 72-78.

Yanisch-Perron, C., Vieira, J., and Messing, J. (1985) Improved M13 phage cloning vectors and host strains: nucleotide sequences of the M13mp18 and pUC19 vectors. *Gene* **33**: 103-119.

Yildiz, F.H., and Schoolnik, G.K. (1998) Role of *rpoS* in stress survival and virulence of *Vibrio cholerae*. *J Bacteriol* **180**: 773-784.
